# Research advances in fungal polysaccharides: production, extraction, characterization, properties, and their multifaceted applications

**DOI:** 10.3389/fcimb.2025.1604184

**Published:** 2025-06-09

**Authors:** Jaturong Kumla, Suppasin Thangrongthong, Atsadawut Kaewnunta, Nakarin Suwannarach

**Affiliations:** ^1^ Office of Research Administration, Chiang Mai University, Chiang Mai, Thailand; ^2^ Center of Excellence in Microbial Diversity and Sustainable Utilization, Chiang Mai University, Chiang Mai, Thailand; ^3^ Department of Biology, Faculty of Science, Chiang Mai University, Chiang Mai, Thailand

**Keywords:** bioactive compound, biological properties, bioprocesses, edible fungi, natural polysaccharide

## Abstract

Fungal polysaccharides have demonstrated significant biological potential, including immune stimulation, antioxidant activity, anticancer properties, and anti-inflammatory effects. These properties hold considerable promise for innovative applications across various fields. This study provides a brief review of current findings, based on literature published over the past 15 years on fungal polysaccharides. This includes the production process and various extraction methods, highlighting their distinct advantages and limitations. Additionally, we summarize techniques for purification and characterization, elucidating their biological properties and practical applications in medicine, pharmacology, the food industry, agriculture, and environment. Global patent trends related to fungal polysaccharides are also reviewed. Finally, we discuss challenges and future perspectives related fungal polysaccharides. This article offers valuable insights and enhances the understanding of fungal polysaccharides for researchers, paving the way for further research and applications.

## 1 Introduction

Fungi have played an important role in human history for millions of years. Terrestrial fossil evidence indicates that fungi first appeared around 400 million years ago (Brundrett, 2002). An updated estimation of the global fungi species has been revised and raised to 2.5 million worldwide according to updated molecular data, but only about 155,000 species have been officially identified and described, highlighting the ongoing research in this field (Antonelli et al., 2023). Belonging to the kingdom of Fungi, they are vital in decomposing organic matter within nutrient cycles and are associated with various organisms that help maintain ecosystems (Niego et al., 2023). Certain edible fungi serve as a crucial food source, providing high nutritional value with proteins, vitamins, and essential minerals, making them suitable for consumption to enhance health. Additionally, some of them exhibit medicinal properties, including antimicrobial, anticancer, and immunomodulatory effects (Valverde et al., 2015). In the industrial sector, extracts from edible fungi are utilized in food and beverage production, such as functional foods, brewing beer, fermenting wine, and making bread (Lorenzo et al., 2018). The cultivation of edible fungi holds significant economic value, providing farmers with income opportunities due to the high market demand (Zhang et al., 2014). Edible fungi can also serve as meat substitutes and are a rich protein source in various dishes, making them an attractive option for those reducing meat consumption or following a vegetarian diet (Ayimbila and Keawsompong, 2023). Interestingly, many edible fungi produce bioactive compounds, which are chemicals that exert biological effects on the human body or other living organisms. Examples of bioactive compounds derived from fungi include polysaccharides, triterpenoids, ergosterol, polyphenols, flavonoids, vitamins, minerals, and antibiotic compounds (Valverde et al., 2015; Antonelli et al., 2023).

**Figure 1:**
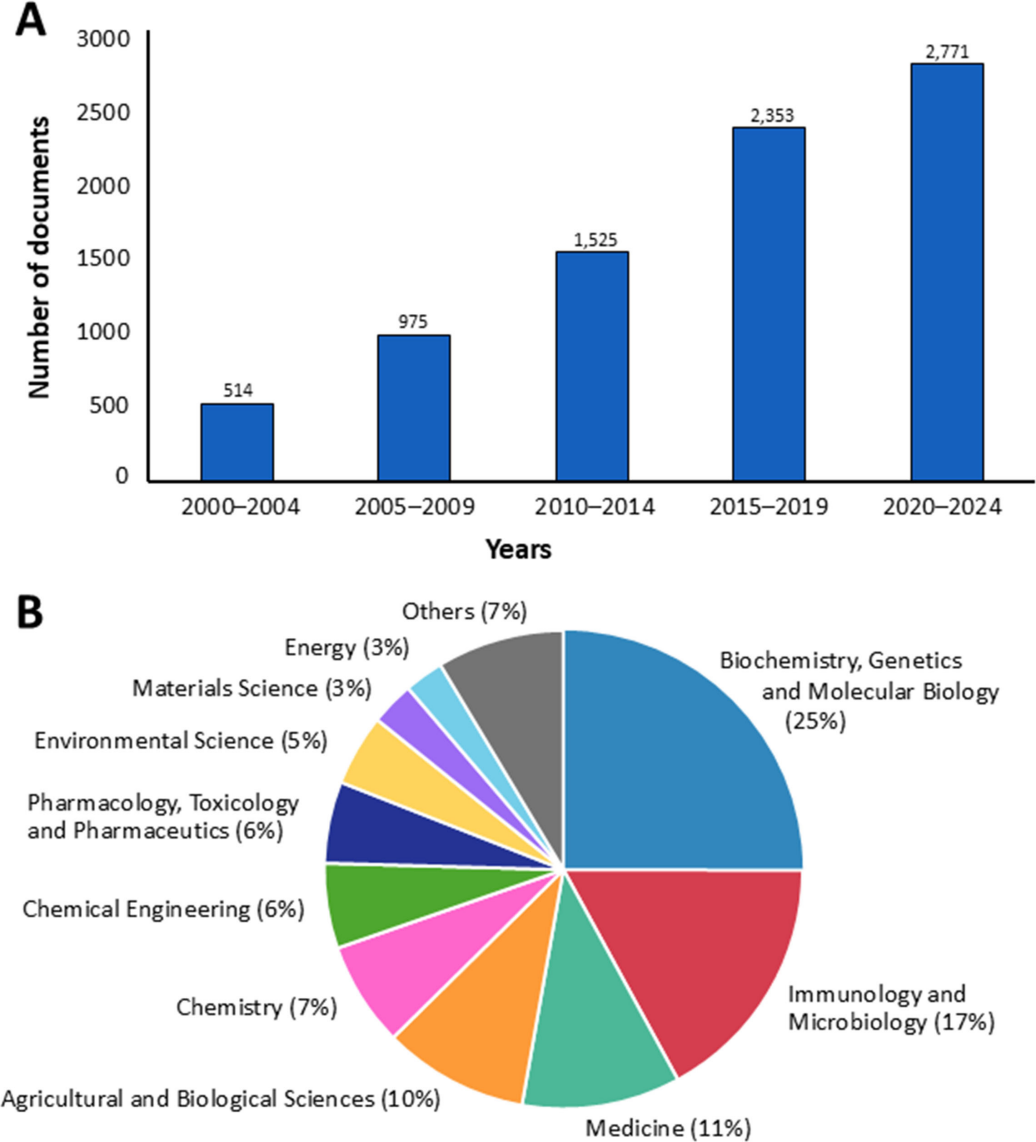
The number of documents **(A)** and the distribution of published research across various subject areas **(B)** on fungal polysaccharides in the Scopus database from 2000 to 2024, based on data from Scopus.

One of the most popular and widely recognized fungal bioactive compounds is polysaccharides. Fungal polysaccharides are complex carbohydrates composed of multiple monosaccharide units linked by glycosidic bonds, and they possess biological properties (Hamidi et al., 2022; Stoica et al., 2023). From 2000 to 2024, a search using the keyword “fungal polysaccharide” retrieved 8,138 titles of documents published over the last 25 years in the Scopus database (https://www.scopus.com, accessed 20 March 2025). It was found that the trend in research on fungal polysaccharides is expected to increase in the future (**Figure 1A**).

**Figure 2:**
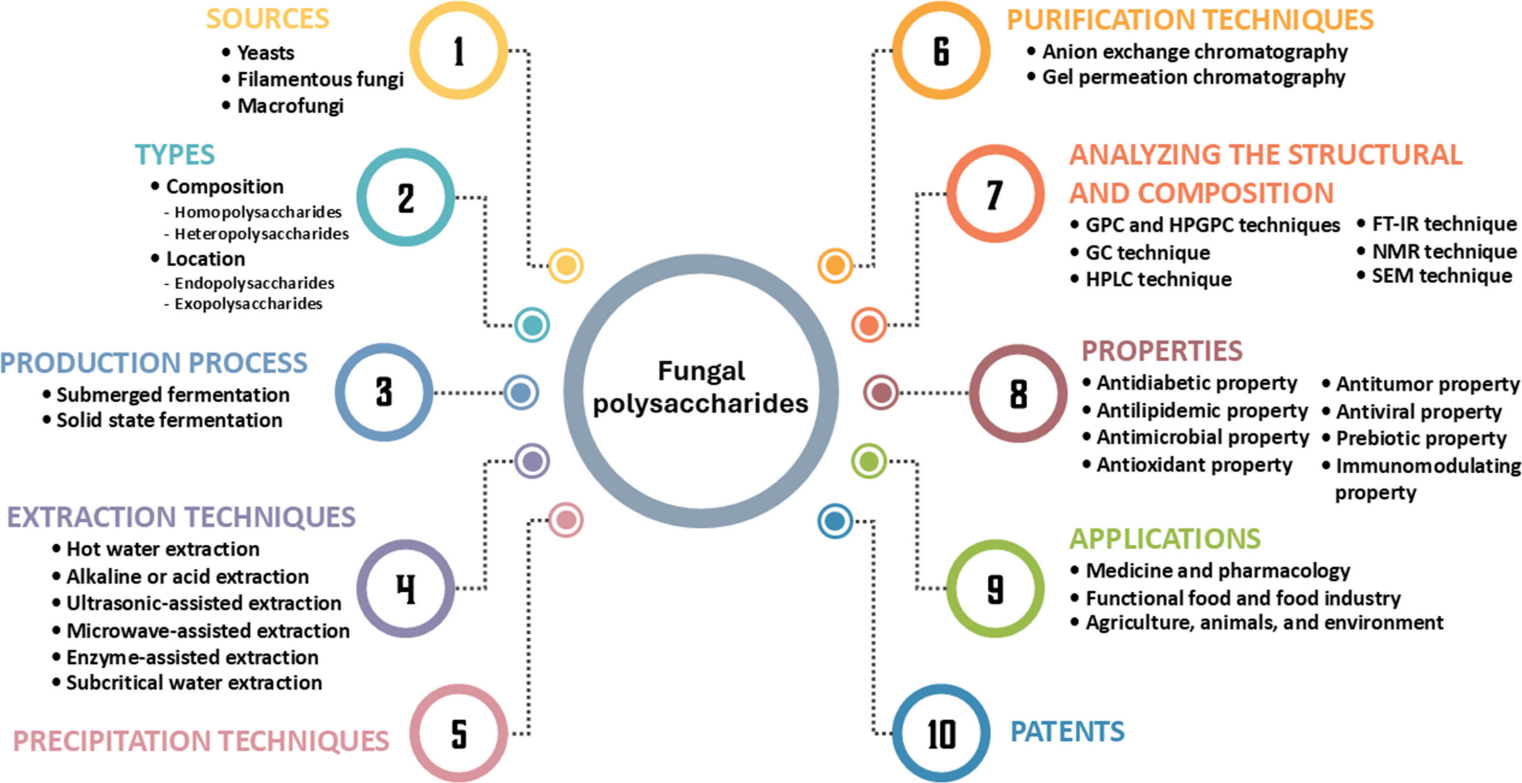
The summarization of this review article.

Additionally, the majority of applications for fungal polysaccharides have been reported in the fields of biochemistry, genetics, and molecular biology (25%), followed by immunology and microbiology (17%), medicine (11%), agricultural and biological sciences (10%), and chemistry (7%) (**Figure 1B**). In this study, we summarize current findings on fungal polysaccharides, including type, production processes, extraction methods, characterization, biological properties, and their applications in various fields from 2010 to 2024 (a 15-year period). Moreover, we summarize trends in patents related to fungal polysaccharides. The overview of this review article is presented in **Figure 2**.

## 2 Source of fungal polysaccharides

Fungal polysaccharides can be produced by yeasts (single cells), filamentous fungi (mycelia), and macrofungi (mycelia and fruiting bodies). Notably, basidiomycetous fungi have attracted significant research attention, particularly for their polysaccharide production and diverse industrial applications. Additionally, the diversity of fungal species and the types of polysaccharides they produce highlight the significant potential of fungi as a source of bioactive compounds for various applications. Examples of fungal species reported to produce polysaccharides are compiled and presented in **Table 1**.

**Table 1:** Some fungal phylum, genus and species used for polysaccharide production over a 15-year period (2010 to 2024).

Phylum	Genus	Species	Reference
Ascomycota	*Aspergillus*	*A*. *fumigatus*, *A*. *medius*, *A*. *oryzae*, *A*. *pseudoglaucus*, and *A*. *versicolor*	Jin and Zhao (2014); Utama et al. (2021); Peng et al. (2023); Wu et al. (2023), and Yang et al. (2024a)
	*Botryosphaeria*	*B. rhodina*	Fonseca et al. (2011) and Miranda-Nantes et al. (2011)
	*Candida*	*C. albicans*, *C. famata*, *C. guilliermondii*, and *C. utilis*	Gientka et al. (2016); Khoury et al. (2020) and Bzducha-Wróbel et al. (2024)
	*Cordyceps*	*Co. cicadae*, *Co. guangdongensis*, *Co. militaris*, and *Co. taii*	Liu et al. (2019); Kang et al. (2022); Yang et al. (2023), and Wang et al. (2024)
	*Cryptococcus*	*Cr. neoformans*	Dornelles et al. (2023)
	*Helvella*	*H. leucopus*	Ge et al. (2022)
	*Kluyveromyces*	*K. lactis* and *K. marxianus*	Backhaus et al. (2010) and Karim et al. (2020)
	*Lasiodiplodia*	*L. theobromae*	Ascencio et al. (2021)
	*Meyerozyma*	*M. guilliermondii*	Wang et al. (2017a)
	*Monascus*	*Mo. kaoliang* and *Mo. purpureus*	Suraiya et al. (2024)
	*Morchella*	*Mor. esculenta* and *Mor*. *sextelata*	Li et al. (2022) and Guo et al. (2024b)
	*Ophiocordyceps*	*O. dipterigena*	Prathumpai et al. (2015)
	*Paecilomyces*	*P. hepiali*	Tian et al. (2024)
	*Penicillium*	*Pe. expansum*, *Pe. janthinellum*, and *Pe. sumatraense*	Scafati et al. (2022); Shao et al. (2023) and Wang et al. (2023a)
	*Pichia*	*Pi. pastoris*	Cheng et al. (2024)
	*Rhizopus*	*R. oryzae*	Anchundia et al. (2024)
	*Saccharomyces*	*S. cerevisiae*	Utama et al. (2021)
	*Schizosaccharomyces*	*Sc. pombe*	Loira et al. (2018)
	*Tuber*	*T. aestivum* and *T. huidongense*	Chen et al. (2016) and Bhotmange et al. (2017)
Basidiomycota	*Agaricus*	*Ag. bisporus*, *Ag. blazei*, and *Ag. brasiliensis*	Zhang et al. (2016); Aguiló-Aguayo et al. (2017), and Li et al. (2020b)
	*Agrocybe*	*Agr. cylindracea*	Liu et al. (2024a)
	*Amauroderma*	*Am. rugosum*	Li et al. (2024b)
	*Antrodia*	*An. cinnamomea*	Lu et al. (2024a)
	*Armillaria*	*Ar. gallica*, *Ar. mellea*, and *Ar. tabescens*	Li et al. (2023); Sun et al. (2023), and Yuchen-Zhang et al. (2024)
	*Aureobasidium*	*Au. melanogenum* and *Au. pullulans*	Elshafie et al. (2017); Wang et al. (2023b), and Tagne et al. (2024)
	*Auricularia*	*Aur. auricula-judae*, *Aur. cornea*, and *Aur. polytricha*	Xiang et al. (2021); Zhou et al. (2023); Zhang et al. (2020), and Lin et al. (2024)
	*Boletus*	*Bo. aereus* and *Bo. auripes*	Luo et al. (2023) and Wei et al. (2024)
	*Butyriboletus*	*Bu. pseudospeciosus*	Tian et al. (2023)
	*Calocybe*	*Cal. indica*	Bains et al. (2023)
	*Calostoma*	*Calo. insigne*	Saengha et al. (2023)
	*Cantharellus*	*Can. cibarius*	Uthan et al. (2021)
	*Chroogomphus*	*Ch. rutilus*	Guo et al. (2024a)
	*Clitocybe*	*Cl. maxima*	Chen et al. (2013)
	*Dictyophora*	*D. indusiata*	Zhang et al. (2023)
	*Exidia*	*E. yadongensis*	Zhong et al. (2024)
	*Flammulina*	*F. velutipes*	Yang et al. (2024d)
	*Fomitiporia*	*Fo. chilensis*	Abdala-Díaz et al. (2024)
	*Fomitopsis*	*Fom. betulina*, *Fom. castane*, *Fom. officinalis*, *Fom. pinicola*, and *Fom. ulmaria*	Zhao et al. (2014); Hao et al. (2016); Jin et al. (2019); Golovchenko et al. (2020); Kizitska et al. (2024)
	*Ganoderma*	*G. applanatum*, *G. lingzhi*, *G. lucidum*, and *G. resinaceum*	Hsu et al. (2017); Si et al. (2018), Chuensun et al. (2021); Mfopa et al. (2021), and Sipping et al. (2022)
	*Grifola*	*Gr. frondosa*	Chen et al. (2019) and Zhang et al. (2018c)
	*Hericium*	*He. coralloides* and *He. erinaceus*	Qin et al. (2017) and Tabibzadeh et al. (2022)
	*Hypsizygus*	*Hy. marmoreus* and *Hy. ulmarius*	Govindan et al. (2023) and Chen et al. (2024)
	*Inonotus*	*I. hispidus* and *I. obliquus*	Chen et al. (2015); Lazić et al. (2024) and Liu et al. (2024b)
	*Lactarius*	*La. deliciosus*, *La. hatsudake*, and *La. quieticolor*	Dong et al. (2024); Yang et al. (2024b), and Zavadinack et al. (2024)
	*Laetiporus*	*Lae. sulphureus*	Jen et al. (2024)
	*Lentinula*	*Le. edodes*	Yin et al. (2018); Zhang et al. (2022a), and Xu et al. (2023)
	*Lentinus*	*Len. lepideus*, *Len. polychrous*, *Len. sajor-caju*, *Len. squarrosulus*, and *Len. velutinus*	Yoon et al. (2011); Udchumpisai and Bangyeekhun (2020); Sakdasri et al. (2022), and Panya et al. (2024)
	*Lepista*	*Lep. sordida*	Wang et al. (2022a)
	*Oudemansiella*	*Ou. raphanipe*	Alitongbieke et al. (2024)
	*Pallidohirschioporus*	*Pa. biformis* (*Trichaptum biforme*)	Habibi et al. (2024)
	*Paxillus*	*Pax. involutus*	Liu et al. (2018)
	*Phellinus*	*Ph. baumii*, *Ph. igniarius*, *Ph. linteus*, and *Ph. rimosus*	Liu et al. (2023a); Ni et al. (2023), and Maingam et al. (2024)
	*Pholiota*	*Pho. adiposa*	Xu et al. (2024)
	*Pleurotus*	*Pl. citrinopileatus*, *Pl. eryngii*, *Pl. ferulae*, *Pl. flabellatus*, *Pl. floridanus*, *Pl. geesterani*, *Pl. ostreatus*, *Pl. pulmonarius*, and *Pl. sapidus*	Duobin et al. (2013); Muthusamy et al. (2013); Wang et al. (2014b); Qiu et al. (2024); Azuar Mohamad et al. (2018); Zhang et al. (2018a); Gil-Ramírez et al. (2019); Rodríguez-Seoane et al. (2022); Lu and Liu (2024), and Rosyida et al. (2024)
	*Rhodototula*	*Rh. minuta* and *Rh. mucilaginosa*	Samadlouie et al. (2020) and Dornelles et al. (2023)
	*Russula*	*Ru. senecis*	Khatua and Acharya (2017)
	*Sanghuangporus*	*Sa. sanghuang* and *Sa. vaninii*	Lu and Liu (2024) and Luo et al. (2024)
	*Schizophyllum*	*Sch. commune*	Chen et al. (2020) and Vanin et al. (2023)
	*Sclerotium*	*Scl. glucanicum* and *Scl. rolfsii*	Fosmer and Gibbons (2011) and Valdez et al. (2021)
	*Sparassis*	*Sp. latifolia*	Lu et al. (2024b)
	*Sporobolomyces*	*Spo. pararoseus* (*Sporidiobolus pararoseus*)	Liao et al. (2024)
	*Stropharia*	*St. rugosoannulata*	Hao et al. (2023a)
	*Suillellus*	*Su. luridus*	Zhang et al. (2018b)
	*Trametes*	*Tr. versicolor*	Angelova et al. (2022)
	*Tremella*	*Tre. fuciformis* and *Tre. sanguinea*	Liu et al. (2023c), and Woźniak et al. (2023)
	*Tricholoma*	*Tri. matsutake* and *Tri. mongolicum*	Yin et al. (2011) and Zhang et al. (2022b)
	*Volvariella*	*V. volvacea*	Cui et al. (2018); Sangthong et al. (2022), and Tepsongkroh et al. (2023)
	*Wolfiporia*	*W. cocos*	Ma et al. (2023)

## 3 Types of fungal polysaccharides

Fungal polysaccharides are categorized primarily into two types based on their composition: homopolysaccharides, composed of a single type of monosaccharide, and heteropolysaccharides, which include various monosaccharides (Murphy et al., 2023a). Homopolysaccharides are polysaccharides composed of only one type of monosaccharide unit repeated in long chains, e.g., glucans (polymers of glucose), chitin (a polymer of N-acetylglucosamine), and mannans (polymers of mannose) (De Felice et al., 2020). Conversely, heteropolysaccharides are polysaccharides composed of two or more different types of monosaccharide units, e.g., galactomannans (composed of mannose and galactose), pectins (composed of galacturonic acid and rhamnose), and glycosaminoglycans (e.g., hyaluronic acid) (Kadooka et al., 2023). Additionally, fungal polysaccharides are broadly divided into two forms based on their cellular location: endopolysaccharides (EnPs) or intracellular polysaccharides, and exopolysaccharides (ExPs) or extracellular polysaccharides (Zhang et al., 2016). These types differ considerably in terms of structure, biological activities, and extraction methods, offering distinct benefits and applications in biotechnological fields.

**Figure 3:**
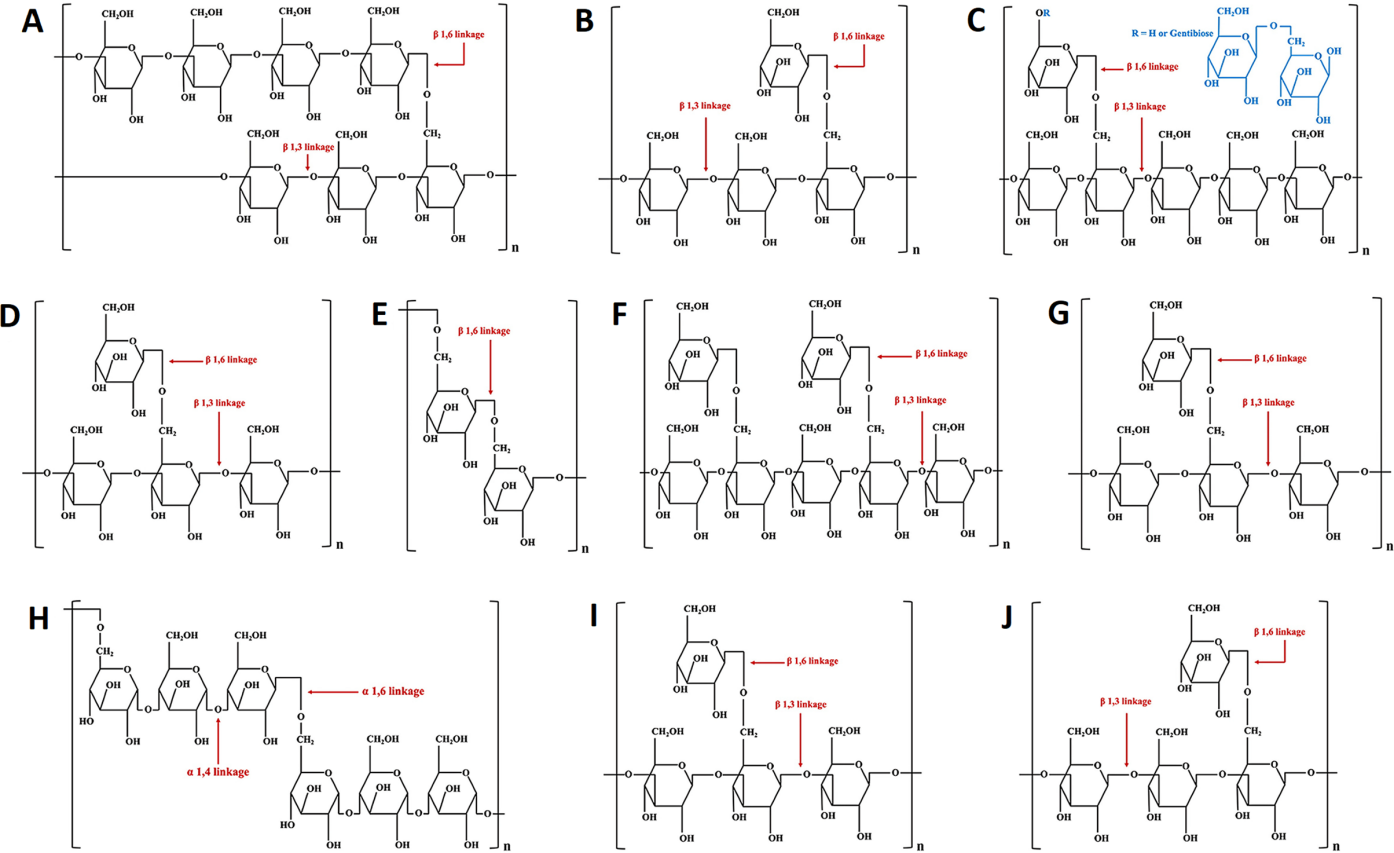
The chemical structures of yeast *β*-glucan **(A)**, fungal *β*-glucan **(B)**, botryosphaeran **(C)**, grifolan **(D)**, lasiodiplodan **(E)**, lentinan **(F)**, pleuran **(G)**, pullulan **(H)**, schizophyllan **(I)**, and scleroglucan **(J)**.

Both fungal EnPs and ExPs are crucial for fungal growth and adaptation to environmental conditions. Fungal EnPs are located within cells and often have complex, branched structures. They are crucial components of cell walls or are stored in the cytoplasm, providing structural support to the cell. They are typically composed of chitin and monosaccharides such as glucose, mannose, and galactose (Wang et al., 2017b; Fernando et al., 2021). *β*-glucan is well-known fungal EnPs, particularly in fungi belonging to the basidiomycetes and ascomycetes groups. In contrast, fungal ExPs are secreted by cells into their surrounding environment, where they generally form linear or mildly branched structures. These structures are typically simpler than those of EnPs. Fungal ExPs contribute to the extracellular matrix, forming gels that facilitate adhesion and biofilm formation, and providing structural support and protection to the fungi under adverse conditions (Mohamed et al., 2024). Primarily composed of glucose, fructose, and other simple monosaccharides, these polysaccharides typically lack complex functional groups and are valued for their gelling and viscosity-enhancing properties (Wang et al., 2020a). Fungal ExPs are often characterized by favorable physical properties such as high-water solubility and significant viscosity, though some types may be insoluble or display unique physical traits based on their structural makeup (Guo et al., 2017). Furthermore, extraction methods for fungal ExPs are usually more straightforward than those for fungal EnPs, as they do not require cell disruption (Jeong et al., 2013; Miao et al., 2020). The most well-known fungal polysaccharides, including both EnPs and ExPs (e.g., botryosphaeran, grifolan, lasiodiplodan, lentinan, pleuran, pullulan, schizophyllan, and scleroglucan), are listed in **Table 2**, and their chemical structures are presented in **Figure 3**.

**Table 2:** Examples of fungal polysaccharides, sources, and types of glycosidic linkages.

Types of Fungal Polysaccharides	Fungal Polysaccharides	Sources	Types of Glycosidic Linkages	References
Endopolysaccharides	Yeast *β*-glucan	*C. albicans*, *K. lactis*, *M. guilliermondii*, *Pi. pastoris*, *S. cerevisiae*, and *Sc. pombe*	*β*-(1,3) with long-branched chains *β*-(1,6)	Utama et al. (2021); Chioru and Chirsanova (2023), and Murphy et al. (2023b)
	Microfungal *β*-glucan	*A. fumigatus*, *A. oryzae*, *Pe. sumatraense*, and *R. oryzae*	*β*-(1,3) and *β*-(1,3) with short-branched chains *β*-(1,6)	Utama et al. (2021); Scafati et al. (2022); Chioru and Chirsanova (2023); Murphy et al. (2023b), and Anchundia et al. (2024)
	Macrofungal *β*-glucan	*Aur. polytricha*, *F. velutipes*, *G. applanatum*, *G. lucidum*, *G. resinaceum*, *Le. edodes*, *Len. lepideus*, *Ph. igniarius*, *Pl. flabellatus*, *Pl. ostreatus*, and *Tr. versicolor*	*β*-(1,3) with short-branched chains *β-*(1,6)	Azuar Mohamad et al. (2018); Cerletti et al. (2021); Chioru and Chirsanova (2023), and Murphy et al. (2023b)
Exopolysaccharides	Botryosphaeran	*B. rhodina*	*β*-(1,3) and *β*-(1,6)	Weng et al. (2011); Geraldelli et al. (2020), and Stoica et al. (2023)
	Grifolan	*Gr. frondose*	*β*-(1,3) and *β*-(1,6)	Hamidi et al. (2022), and Stoica et al. (2023)
	Lasiodiplodan	*L. theobromae*	*β*-(1,6)	Alves da Cunha et al. (2012); Ascencio et al. (2021); Hamidi et al. (2022); Wouk et al. (2022), and Stoica et al. (2023)
	Lentinan	*Le. edodes*	*β*-(1,3) and *β*-(1,6)	Ren et al. (2018); Hamidi et al. (2022); Stoica et al. (2023), and Xu et al. (2023)
	Pleuran	*Pl. ostreatus*	*β*-(1,3) and *β*-(1,6)	Jesenak et al. (2016); Hamidi et al. (2022), and Urbancikova et al. (2020)
	Pullulan	*Au. pullulans*, *Au. melanogenum*, *Cry. parasitica*, *Rh. bacarum*, and *Tre. mesenterica*	*α*-(1,6) and *α*-(1,4)	Hamidi et al. (2022); Stoica et al. (2023); Thakur et al. (2023); Wang et al. (2023b), and Tagne et al. (2024)
	Schizophyllan	*Sc. commune*	*β*-(1,3) and *β*-(1,6)	Mansour et al. (2012); Hamidi et al. (2022), and Stoica et al. (2023)
	Scleroglucan	*Scl. rolfsii* and *Scl. glucanicum*	*β*-(1,3) and *β*-(1,6)	Viñarta et al. (2013); Valdez et al. (2021); Bai et al. (2022); Hamidi et al. (2022), and Stoica et al. (2023)

## 4 Production process of fungal polysaccharides

The industrial-scale production of fungal polysaccharides from fungi is a process that requires precise control and high efficiency to ensure the desired quantity and quality of the product. The production process begins with the selection of fungal strains capable of producing large amounts of polysaccharides (Sánchez et al., 2015; Junior Letti et al., 2018). Additionally, inoculum preparation under optimal conditions is crucial to maximize fungal biomass before transferring to the fermentation system. The choice of fermentation method is critical, with the primary approaches being submerged fermentation and solid state fermentation (Bakratsas et al., 2021). Solid state fermentation occurs on solid substrates or non-soluble support materials and is particularly suitable for certain fungal fruit bodies (Junior Letti et al., 2018), utilizing agricultural residues as growing substrates (Pérez-Chávez et al., 2019). In contrast, submerged fermentation, a common industrial method, involves liquid-based fermentation requiring the careful control of parameters such as pH, temperature, agitation, and aeration (Sánchez et al., 2015). The enhancement of polysaccharide production from both fermentation processes requires optimal conditions for fungal growth specific to each fungal species and strain (Montoya et al., 2013).

### 4.1 Solid state fermentation

Solid state fermentation (SSF) involves the cultivation of fungi on solid substrates or low-moisture materials, typically derived from agricultural or industrial residues such as wheat straw, rice straw, coffee pulp, wood logs, sawdust, bran, husks, and sugarcane bagasse (Junior Letti et al., 2018; Yafetto, 2022), due to the ability of fungi to break down and utilize lignocellulosic materials as a source of nutrients to support their growth and development. Additionally, the selection and utilization of agricultural or industrial residues in SSF is contingent on their availability in each country. SSF is particularly suitable for cultivating fungi to produce their fruiting bodies, commonly known as mushrooms (Junior Letti et al., 2018). The cultivation of mushrooms using SSF has been studied worldwide because it allows the use of various agricultural or industrial residues as substrates, promotes the recycling of these residues into materials for fungal growth, and facilitates their conversion into fruiting bodies (Grimm and Wösten, 2018; Junior Letti et al., 2018). For example, Sánchez and Montoya (2020) found that the cultivation of *Pl. ostreatus*, *Le. edodes*, and *Tr. versicolor* under SSF conditions using oak sawdust revealed that the highest polysaccharide production was recorded for the growing substrate of *Tr. versicolor* (96.09 mg/g of solid substrate), followed by *Pl. ostreatus* (90.78 mg/g) and *Le. edodes* (87.45 mg/g). The research conducted by Xu et al. (2019) found that the optimal conditions for polysaccharide production from *Co. militaris* using rice under SSF resulted in a maximum yield of 68.3 mg/g of dry substrate. Shi et al. (2012) used soybean curd residue as a substrate in SSF to produce polysaccharides from the fruiting bodies of *F. velutipes*, yielding 106.74 mg/g of dried fruiting bodies. Additionally, *Le. edodes* fruiting bodies cultivated under SSF using beechwood sawdust yielded a maximum polysaccharide production of 67.33 mg/g of dry weight (Reza et al., 2024). SSF of *Gr. frondosa* using corn bran and oak sawdust resulted in a polysaccharide yield of 60.5 mg/g of dry weight (Montoya Barreto et al., 2011). Thus, SSF can be used for the production of fungal polysaccharides, demonstrating its economic viability as well as its potential for further pilot research and large-scale industrial applications. Remarkably, the carbon/nitrogen (C/N) ratio and the composition of cellulose, hemicellulose, and lignin in agricultural or industrial substrates vary across different types, making them crucial factors in SSF. Large-scale SSF, particularly in industrial settings, remains challenging due to the need to carefully control factors such as pH, heat and mass transfer, water activity, fungal strain selection, substrate heterogeneity, C/N ratio, and optimal moisture levels for fungal growth.

### 4.2 Submerged fermentation

Submerged fermentation involves the cultivation of fungal mycelium in a liquid medium, where nutrients are dissolved, and agitation is used to facilitate aeration within the fermenter or bioreactor. The primary advantages of submerged fermentation include efficient oxygen transfer and homogeneous distribution of the liquid medium. However, this technique requires meticulous control of various factors such as nutrient (carbon and nitrogen sources), temperature, aeration, agitation, pH, medium composition, and the type and amount of inoculum. Consequently, submerged fermentation is a reproducible technique for the continuous cultivation of fungal mycelium, enabling controlled production of metabolites (Elisashvili, 2012). Nonetheless, prolonged cultivation in liquid media can lead to increased medium viscosity due to the growth and accumulation of fungal mycelium, potentially impacting oxygen distribution, carbon dioxide removal, and product dilution (Bentil et al., 2018). Recently, edible fungi, particularly those from the basidiomycetes and ascomycetes genera, including *Agaricus*, *Cordyceps*, *Ganoderma*, *Lentinus*, and *Pleurotus*, have been successfully cultivated using bioreactors (Bakratsas et al., 2021). For example, Wang et al. (2019a) achieved the highest extracellular polysaccharide yield (5.713 g/L) of *Co. militaris* in a 5-L bioreactor using glucose and yeast extract as carbon and nitrogen sources, with conditions of 25°C, 150 rpm shaking speed, and 1.5 vvm aeration. The most suitable submerged fermentation conditions for *Le. crinitus* to achieve the highest yield of ExPs at 0.65 g/L in a 5-L bioreactor, using Kirk’s liquid medium, were 30°C, pH 4.5, 300 rpm stirring, and a 1.5 vvm aeration rate for four days (López-Legarda et al., 2020). The fed-batch submerged fermentation process for EnP production from *G. lucidum* was successfully scaled up in stages, from 7.5 L to 20 L, and finally to a 200-L stirred-tank reactor, where maintaining a low impeller tip speed of 1.234 m/s resulted in a maximum EnP production of 4.74 g/L (Tang et al., 2011). Moreover, Wu et al. (2013) studied the effect of different light wavelengths on ExPs production from *Pl. eryngii* in submerged cultivation and found that ExPs production was highest under blue light (455 mg/L) condition, compared to green (425 mg/L), red (217 mg/L), yellow (314 mg/L), white (50 mg/L), and dark (59 mg/L) conditions. In a recent study by Bakratsas et al. (2024), the cultivation of *Pl. ostreatus* was successfully scaled up in a 3.5 L bioreactor under optimal conditions, resulting in a maximum biomass of 12.6 g/L, the highest ExPs production of 3.7 g/L.

Overall, previous studies highlight several key factors that significantly enhance the production efficiency of polysaccharides from selected fungi. Among these factors, the selection of suitable carbon sources, such as glucose and yeast extract, plays a crucial role in stimulating fungal growth and the production of desired compounds. Optimal concentrations of nutrients should be maintained to support maximum biomass development and polysaccharide yield. Additionally, controlling fermentation parameters such as temperature, pH, agitation, and aeration is crucial for optimizing polysaccharide production. Furthermore, the use of specific light wavelengths during cultivation has been shown to enhance the production of fungal ExPs.

## 5 Extraction techniques for fungal polysaccharides

**Figure 4:**
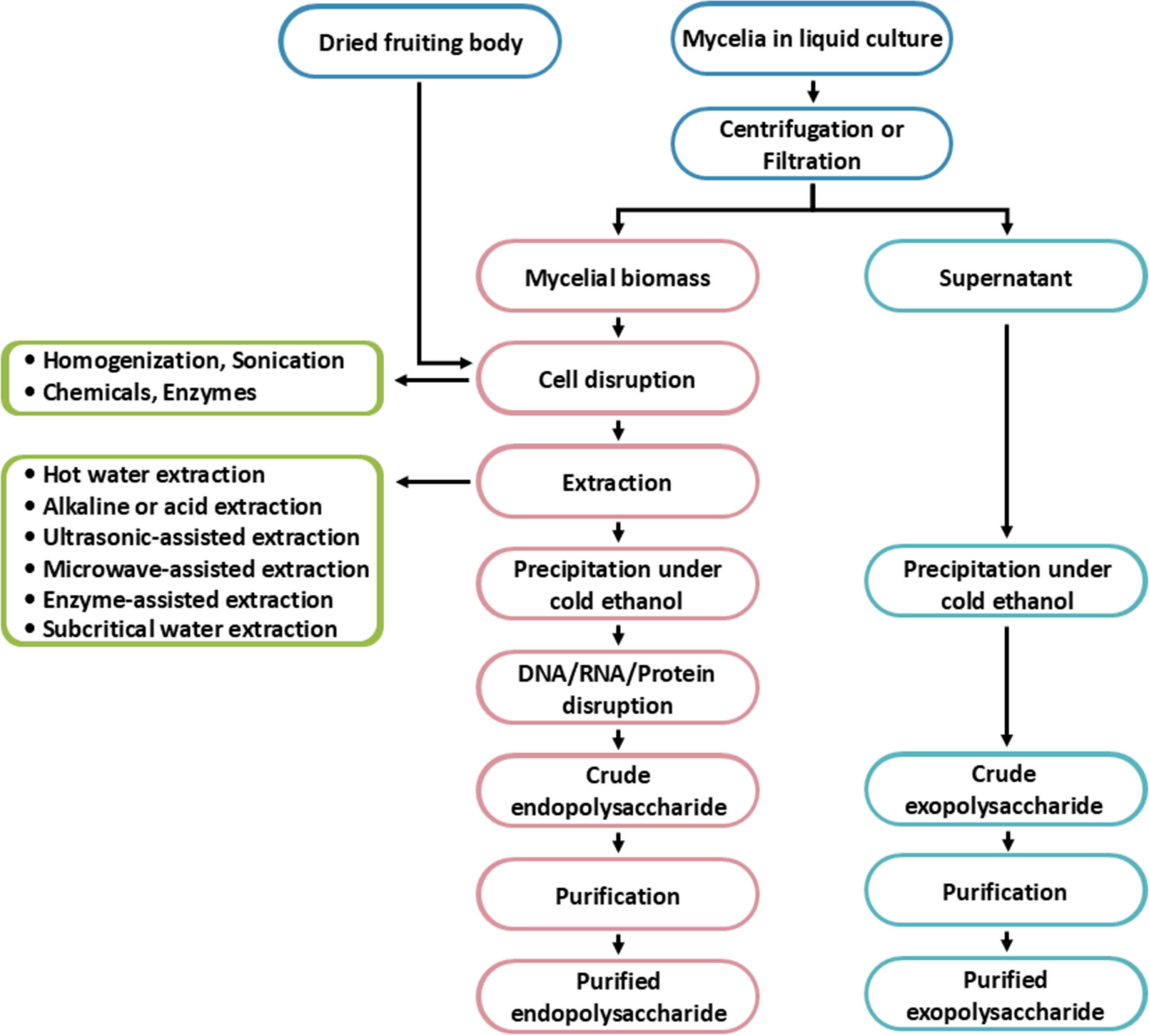
The process of fungal endopolysaccharides and exopolysaccharides production.

The processes for obtaining fungal EnPs and ExPs involve several steps, as shown in **Figure 4**. The extraction of polysaccharides from fungi primarily differs in the locations of the polysaccharides and the methods employed for extraction. For EnP extraction, the process begins with harvesting the fungal mycelia or fruiting bodies, followed by cell disruption, which can be achieved either chemically or mechanically to break the cell walls (Wang et al., 2015b; Liu et al., 2022b). On the other hand, ExP is secreted outside the fungal cells and accumulates in the liquid culture or surrounding fluid. The extraction of ExPs typically involves filtering or separating the liquid culture from the fungal biomass. The ExP extraction is simpler because it does not require cell disruption. As a result, ExPs tend to be purer since they are directly extracted from the fungal supernatant (Jeong et al., 2013). However, EnP extraction could be contaminated by other intracellular substances, requiring further purifying processes.

### 5.1 Extraction of fungal exopolysaccharides

Generally, submerged fermentation is used for fungal ExP production. The extraction of ExP typically involves straightforward methods. Fungal ExP is separated from the liquid culture medium by first removing the fungal mycelia and cells through filtration or centrifugation and then collecting the supernatant (Stoica et al., 2023). The ExP present in the supernatant is then precipitated with organic solvents (e.g., acetone, ethanol, propanol, or isopropyl alcohol) under cold conditions (Maziero et al., 1999; Elisashvili et al., 2009). After precipitation, centrifugation or filtration is used to collect the crude fungal ExP.

### 5.2 Extraction of fungal endopolysaccharides

The EnP extraction from fungi is a more complex procedure than ExP extraction and requires specific methods to isolate EnP from the fungal cells (Jeong et al., 2013). After the fungus cells or fruiting bodies have been collected, the cell walls must be broken down in order to release the EnP. This can be achieved through chemical techniques, such as using lysozyme enzymes or solvents that degrade the fungal cell walls, or mechanical techniques like grinding or ultrasonic waves to break down the cell walls (Ferraboschi et al., 2021; Larrañaga-Ordaz et al., 2022). After the cell walls are disrupted, the extraction process can be carried out using a variety of techniques, including hot water extraction, alkaline- or acid-extraction, ultrasonic-assisted extraction, microwave-assisted extraction, enzyme-assisted extraction, or subcritical liquid extraction. Following extraction, EnP is collected, purified, and freeze-dried for subsequent application (Leong et al., 2021; Zhao et al., 2023b). Examples of fungi used for EnP extraction through various techniques are shown in **Table 3**.

**Table 3:** Examples of EnP extraction from some fungi.

Fungal Species	Extraction Techniques*	Temperature (°C)	Time (min)	Yield (g of 100 dried mycelia or fruiting bodies)	Reference
*Agaricus bisporus*	AE (1 M NaOH)	50	360	4.31	Li et al. (2017a)
	EAE (pH 4, papain: pectinase: cellulase = 1:1:1)	62	180	6.87	Yin et al. (2015)
	UAE (230 W ultrasonic power)	70	62	6.02	Tian et al. (2012)
	UAE (400 W ultrasonic power)	RT	15	4.70	Aguiló-Aguayo et al. (2017)
*Auricularia auricula*	MAE (860 W microwave power)	95	25	95.8	Zeng et al. (2012)
*Auricularia polytricha*	UMAE (59 W microwave power, 50 W ultrasonic power at a frequency of 40 KHz)	NR	15.3	4.10	Zhang et al. (2020)
*Calocybe indica*	EAE (pH 4.64, cellulase: pectinase: protease = 1:1:1)	47	120	7.24	Bains et al. (2023)
*Cordyceps militaris*	HWE	90 to 95	180	91.2	Liu et al. (2024c)
	SWE	180	13	7.13	Luo et al. (2017)
*Dictyophora indusiata*	UAME (150 W microwave power, 550 W ultrasonic power)	RT	6	12.66	Zhang et al. (2023)
*Flammulina velutipes*	AE (2% KOH)	100	150	9.71	Kawahara et al. (2016)
	HWE	91	120	3.74	Hao et al. (2023b)
	UAE (150 W ultrasonic power)	55	90	109.54	Yang et al. (2024d)
*Fomitopsis ulmaria*	MAE (400 W microwave power)	NR	2.5	8.36	Zhao et al. (2014)
*Ganoderma applanatum*	HWE	100	180	2.14	Mfopa et al. (2021)
*Ganoderma lucidum*	MAE (800 W microwave power)	NR	1.30	13.08	Chuensun et al. (2021)
	UAE (210 W ultrasonic power)	80	100	0.63	Zheng et al. (2020)
*Grifola frondosa*	HWE	95	360	9.1	Zhang et al. (2018c)
	UAE (500 W ultrasonic power)	90	71	21.72	Liu et al. (2023b)
*Helvella leucopus*	HWE	95	240	30.07	Ge et al. (2022)
*Hericium erinaceus*	EAE (pH 5.7, cellulase: pectinase: papain = 2:1:1)	50	79	13.9	Qin et al. (2017)
	EAE (pH 5.7, cellulase: pectinase: trypsin = 2:2:1)	52.03	33.79	13.46	Zhu et al. (2014)
*Inonotus obliquus*	EAE (pH 4.5, viscozyme L, 5% enzyme conc.)	50	120	5.86	Hwang et al. (2019)
	MAE (470 W microwave power, 50 Hz)	NR	30	385.98	Lazić et al. (2024)
	SWE	200	13.24	14.14	Ma et al. (2024)
	UAE (139 W ultrasonic)	42	120	3.02	Hwang et al. (2019)
*Lentinula edodes*	AE (NaOH, 0.1 mol/L)	60	120	95.8	Li et al. (2019a)
	EAE (pH 5, papain: pectinase: cellulase = 4:3:3)	54	93	15.65	Zhao et al. (2016b)
	MAE (850 W microwave power, 2,455 MHz)	180	30	15.4	Gil-Ramírez et al. (2019)
	SWE (0.15 MPa pressure)	115	80	8.20	Li et al. (2018a)
	SWE (1.5 MPa pressure)	140	20	5.25	Zhang et al. (2019)
	UAE (340 W ultrasonic power)	25	14	14.39	Ke (2015)
*Lentinus polychrous*	HWE	90	240	6.77	Panya et al. (2024)
*Lentinus squarrosulus*	HWE	100	180	2.48	Ayimbila et al. (2021)
	HWE	90	240	7.14	Panya et al. (2024)
*Morchella sextelata*	SWE (1 MPa pressure)	153	17	18.09	Li et al. (2022)
*Paxillus involutus*	HWE	79	180	12.25	Liu et al. (2018)
*Peurotus ostreatus*	AE (1 M NaOH)	100	1440	4.5	Palacios et al. (2012)
*Pleurotus citrinopileatus*	SWE	190	NR	16.26	Rodríguez-Seoane et al. (2022)
*Pleurotus eryngii*	HWE	100	360	7.0	Abreu et al. (2021)
	SWE	190	NR	47.57	Rodríguez-Seoane et al. (2022)
*Pleurotus ostreatus*	HWE	90	30	30.95	Rosyida et al. (2024)
	MAE (450 W microwave power)	85 to 90	30	61.73	Rosyida et al. (2024)
	SWE (4 MPa pressure)	180	20	20.35	Rizkyana et al. (2022)
	UAE (550 W ultrasonic power)	RT	30	23.25	Rosyida et al. (2024)
*Pleurotus pulmonarius*	MAE (850 W microwave power)	180	30	15.4	Gil-Ramírez et al. (2019)
*Lentinus sajor-caju*	HWE (Pressure 0.92 MPa)	140	40	3.20	Sakdasri et al. (2022)
*Russula senecis*	AE (10% NaOH)	4	1440	9.71	Khatua and Acharya (2017)
*Schizophyllum* *commune*	HWE	100	120	8.26	Chen et al. (2020)
	HWE	121	120	5.95	Saetang et al. (2022)
*Tricholoma mongolicum*	AE (0.1 M HCl)	90	60	10.83	Zhang et al. (2022b)
	AE (0.1 M NaOH)	90	60	13.16	Zhang et al. (2022b)
	EAE (pH 4, cellulase, 2% enzyme conc.)	50	127	18.96	Zhao et al. (2016a)
*Tuber aestivum*	EAE (pH 6, trypsin: pectinase: papain = 1:2:1)	50	90	46.93	Bhotmange et al. (2017)
*Volvariella volvacea*	HWE and HPP (600 MPa pressure, 10 min pressure time)	60	2	12	Tepsongkroh et al. (2023)
	MAE (860 W microwave power)	95	25	11.05	Sangthong et al. (2022)
	UAE (175 W ultrasonic power)	57	33	8.28	Cui et al. (2018)
*Wolfiporia extensa*	EAE (pH 5, α-amylase and cellulase)	40	180	4.14	Ashfaque et al. (2017)

*HWE, Hot water extraction; AE, Alkaline- or acid-extraction; UAE, Ultrasonic-assisted extraction; MAE, Microwave-assisted extraction; EAE, Enzyme-assisted extraction; SWE, Subcritical water extraction; UAME, Ultrasonic-microwave-assisted extraction; and HPP, High-pressure processing; RT, room temperature; NR, not reported.

#### 5.2.1 Hot water extraction

The hot water extraction method is a popular technique for EnP extraction from fungi due to its simplicity, safety, and the availability of common materials (Dai et al., 2023). The fungal cells or fruiting bodies are collected, dried, and ground into a fine powder to enhance extraction efficiency. By boiling the powdered fungal samples in hot water at temperatures between 50°C and 121°C for 30 min to 10 h, the cell walls are broken down, releasing the EnPs (Saetang et al., 2022). The resulting solution is then filtered to remove impurities, and alcohol is added to concentrate and precipitate the EnP. The EnP is subsequently collected and dried (Leong et al., 2021). This method is suitable for various fungi and can be carried out in a standard laboratory, provided that temperature and extraction time are carefully controlled to achieve optimal results (Parniakov et al., 2014; Leong et al., 2021). The advantages of this method include the absence of toxic chemicals and low cost; however, some loss of EnP may occur due to heat, and the extraction may be incomplete if the cell walls are not fully broken down.

#### 5.2.2 Alkaline or acid extraction

The alkaline or acid extraction technique involves using alkaline or acidic solutions to break down the cell walls of fungi and release the EnP. In alkaline extraction, an alkaline solution, such as sodium hydroxide (NaOH) or potassium hydroxide (KOH), is used to break down the fungal cells. In contrast, an acidic solution, such as hydrochloric acid (HCl) or ammonium oxalate [(NH_4_)_2_C_2_O_4_], is used in acid extraction. After the extraction process, the extracted samples must be pH-neutralized before filtration and precipitation of the polysaccharide (Yi et al., 2020; Leong et al., 2021). Alkaline extraction, using NaOH as the extracting agent, is more commonly employed than acid extraction for isolating fungal polysaccharides. NaOH solutions are typically used in concentrations ranging from 0.1 M to 1 M, with extraction ratios varying from 1:5 to 1:20 (*w/v*) (Palacios et al., 2012; Yang et al., 2019). Nevertheless, acid extraction methods remain an alternative, with previous reports highlighting the use of 0.1 M HCl (1:10 *w*/*v*) and 1% (*w*/*v*) (NH_4_)_2_C_2_O_4_ for extraction (Sermwittayawong et al., 2018; Zhang et al., 2022a). This technique is highly effective, especially for extracting EnPs from fungi with robust cell walls. However, careful control of extraction conditions is necessary to prevent EnP degradation and avoid side effects from the use of highly concentrated alkaline or acidic solutions (Wang et al., 2015b, c).

#### 5.2.3 Ultrasonic-assisted extraction

Ultrasonic-assisted extraction is a method that uses ultrasonic waves to enhance the extraction of EnP from fungal cells. This technique involves using high-frequency sound waves to create cavitation, a phenomenon where small bubbles in the liquid rapidly collapse, producing energy that can break down the cell walls of mycelia or mushrooms and facilitate the release of EnP into the extraction solvent. The ultrasonic technique can be divided into two types: low-intensity and high-intensity ultrasound. The low-intensity ultrasonic technique uses frequency waves ranging from 5 to 10 MHz and energy levels of less than 1 W/cm². On the other hand, the high-intensity ultrasonic technique uses frequencies ranging from 20 to 100 kHz and higher energy levels from 10 to 1,000 W/cm². In comparison to the low-intensity technique, the high-intensity ultrasonic method has a higher destructive capability (Charoux et al., 2017; Li et al., 2024a). In general, EnP extraction using this technique typically involves ultrasonic treatment for 14 to 180 min at temperatures ranging from approximately 25°C to 95°C (Ke, 2015). The process begins by preparing the fungal mycelia or mushroom samples, grinding or cutting them into small pieces, immersing them in an appropriate solvent, and applying ultrasonic waves to facilitate the extraction. Subsequently, the extracted sample is filtered to separate the solid residues, and the solution containing EnP is then precipitated and the EnP is harvested. The advantages of this technique include increased extraction efficiency and reduced solvent usage (Leong et al., 2021; Shen et al., 2023).

#### 5.2.4 Microwave-assisted extraction

Microwave-assisted extraction is a technique that uses microwave energy to accelerate the extraction process. Microwave energy causes water molecules or solvents within the sample to vibrate, generating heat that helps break down the cell structure and release the desired compounds more efficiently (Hu et al., 2021). Key factors influencing EnP yield using this method include microwave power, typically ranging from 400 to 1200 W, extraction temperature, maintained between 85°C and 180°C, and extraction time, which ranges from 1 to 30 min (Zhao et al., 2014; Gil-Ramírez et al., 2019; Chuensun et al., 2021). This technique is a highly efficient and fast technique that reduces solvent and energy usage (Chen et al., 2013). However, it has limitations, including high initial costs, restrictions on solvent selection, risks of unwanted reactions, and challenges in controlling various parameters (Xu et al., 2018).

#### 5.2.5 Enzyme-assisted extraction

Enzyme-assisted extraction is a technique that uses enzymes to extract EnP from fungi. This method leverages the ability of enzymes to break down the cell walls of fungi (Leong et al., 2021; Bains et al., 2023). Several cellulolytic enzymes and proteolytic enzymes have been used, including cellulase, papain, pectinase, protease, and trypsin (Yin et al., 2015; Bhotmange et al., 2017; Bains et al., 2023). The process begins with sample preparation, where the fungal materials are ground or milled into a fine powder. The powder is then mixed with an enzyme solution under optimized conditions to maximize enzyme activity, typically at a pH of 4 to 6, a temperature of 40°C to 60°C, and an incubation time of 30 to 180 min (Zhu et al., 2014; Yin et al., 2015; Ashfaque et al., 2017; Bhotmange et al., 2017). The enzyme is allowed to act for a period during which the cell walls are degraded, and the polysaccharides are released into the solution. After extraction, the solution is filtered, and the polysaccharides are precipitated and harvested (Qin et al., 2017; Bains et al., 2023). This technique offers advantages such as increased extraction efficiency and reduced use of harsh chemicals, making it an environmentally friendly method (Chen et al., 2013; Zhao et al., 2016b). However, it also has disadvantages, including high costs, enzyme stability that depends on environmental conditions, challenges in selecting appropriate enzymes, potentially longer extraction times, and the complexity of process optimization (Yin et al., 2011; You et al., 2013).

#### 5.2.6 Subcritical water extraction

Subcritical water extraction is a technique that uses subcritical fluids to extract polysaccharides from fungi. A subcritical fluid is a liquid heated above its boiling point (100 to 374°C) but remains in liquid form due to the increased pressure (1 to 22.1 MPa) (Gbashi et al., 2017). This technique typically uses water, ethanol-water mixtures, or other solvents (ethanol, methanol, or acetone) under subcritical conditions for extraction (Herrero et al., 2006). The process begins by preparing the fungal material, which is then mixed with the subcritical fluid in a closed system where both temperature and pressure are carefully controlled (Morales et al., 2019; Leong et al., 2021). In subcritical conditions, the physical properties of the fluid, such as its solubility and diffusivity, change, which enhances the efficiency of polysaccharide extraction from fungal cells. After extraction, the solution is cooled and separated to isolate the desired polysaccharides, while the remaining fluid can be reused in subsequent processes (Zhang et al., 2022a). IPS extraction using this technique requires optimization of conditions, typically involving pressures ranging from 0.15 to 5 MPa, temperatures between 115°C and 210°C, and extraction durations of approximately 13 to 80 min (Luo et al., 2017; Li et al., 2018a; Zhang et al., 2019). This technique offers advantages, including shorter extraction times, reduced solvent usage, and better control over the quality of the extracted polysaccharides (Huber et al., 2021). Despite its benefits, this technique has some limitations. One challenge is the need for equipment that can withstand high pressure and temperature, resulting in higher installation costs. Furthermore, operating at high pressure requires specialized expertise to manage the process effectively (Yabalak et al., 2024).

Several previous studies have compared different extraction techniques to obtain the highest yield of fungal EnPs. For example, Sangthong et al. (2022) compared ultrasonic-assisted extraction (sonication at 95°C for 5 h), and microwave-assisted extraction (860 W for 25 min) with hot water extraction (95°C for 5 h) and found that hot water extraction yielded higher EnP levels from *V. volvacea* (15.58% of dried fruiting bodies) compared to microwave-assisted extraction (11.05%) and ultrasonic-assisted extraction (9.06%). Similarly, Chen et al. (2020) compared ultrasonic-assisted extraction (450 W for 20 min) and microwave-assisted extraction (550 W for 5 min) with hot water extraction (100°C for 2 h) and found that hot water extraction yielded the highest polysaccharide content from *Sch. commune* (8.26% of dried fruiting bodies), higher than both ultrasonic-assisted extraction (5.07%) and microwave-assisted extraction (4.98%). Additionally, Zhang et al. (2022b) conducted a comparative study on five extraction methods for isolating EnP from fruiting bodies of *Tri. mongolicum*, including hot water extraction (85°C for 3 h), ultrasonic-assisted extraction (300 W power at 60°C for 35 min), enzyme-assisted extraction (using 5% cellulase and 2% pectinase at pH 5 and 50°C for 100 min), alkaline extraction (0.1 M NaOH at 90°C for 1 h), and acid extraction (0.1 M HCl at 90°C for 1 h). Among these five methods, alkaline extraction yielded the highest polysaccharide content at 13.16% of dried fruiting body, followed by acid extraction (10.83%), hot water extraction (6.64%), enzyme-assisted extraction (5.87%), and ultrasonic-assisted extraction (4.41%). Therefore, the selection of an extraction method for polysaccharides from fungi depends on the characteristics of the fungal samples, the equipment, and the limitations of each extraction technique. Each extraction method has its own distinct advantages and limitations, as summarized in **Table 4**. Hot water extraction is a convenient and safe method, although it may result in partial loss of polysaccharides due to the heat and may not efficiently extract them if the fungal cell walls are not adequately disrupted. For fungi with hard cell walls, alkaline or acid extraction works quite well. However, extreme acidity or alkalinity must be avoided to prevent polysaccharide degradation. Ultrasonic-assisted and microwave-assisted extractions enhance efficiency by utilizing energy from sound waves or microwaves; however, these methods require careful control of energy and extraction time, as they are costly and may limit solvent selection. In addition, enzyme-assisted extraction is effective for breaking down fungal cell walls, but it requires costly enzymes and may involve longer process optimization times. Lastly, subcritical water extraction, which operates at temperatures below the boiling point of water, is efficient and fast; however, it is costly because it requires specialized equipment capable of withstanding high temperatures and pressures.

**Table 4:** Comparison of the principles, key factors, advantages, and limitations of fungal polysaccharide extraction techniques.

Extraction Techniques	Principle	Key Factors	Advantages	Limitations
Hot water extraction	Hot water incubation of fungi at 50-100°C for 1–5 hours helps break down fungal cell walls	Treatment temperature and extraction time	Simple to execute, low-cost, and non-toxic chemicals	Some loss of essential compounds occurs due to heat and the cell walls of certain types of fungi may not be completely broken down
Alkaline- or Acid-extraction	Alkaline or acidic solutions break down fungal cell walls	Treatment temperature and extraction time	Effective with fungi that have robust cell walls	Intensive use of chemicals and not environmentally friendly
Ultrasonic-assisted extraction	Ultrasonic waves create cavitation, causing rapid bubble collapse that disrupts fungal cell walls	Treatment temperature, extraction time, liquid-solid ratio, ultrasonic power, and frequency	Reduces solvent usage, decreases extraction time and resource consumption, and environmentally friendly	Difficult to control temperature and requires adjustment of energy levels or frequency
Microwave-assisted extraction	Microwave energy accelerates extraction by heating vibrating water molecules and breaking cell structures	Treatment temperature, extraction time, and microwave power	Easy to perform, reduces time, and decreases solvent and energy consumption	Risk of unwanted reactions and the chemical structure of key compounds may be compromised
Enzyme-assisted extraction	Enzymes degrade the fungal cell wall by breaking down chitin, cellulose, or cell wall proteins.	Treatment temperature, extraction time, liquid-solid ratio, pH value, type of enzyme, and concentration and ratio of enzyme	High specificity and efficiency, reduces the use of harsh chemicals, and environmentally friendly	High cost and enzyme stability depend on environmental conditions
Subcritical water extraction	Subcritical fluids, kept above their boiling point but in liquid form under pressure, breaking cell structures	Treatment temperature, extraction time, liquid-solid ratio, frequency, and pressure	Reduces processing time, decreases solvent usage, and environmentally friendly	High cost and requires equipment that can withstand high pressure and temperature

Interestingly, the combination of extraction techniques can enhance polysaccharide yields, resulting in higher yields compared to using a single technique. For instance, Zhang et al. (2023) demonstrated that the combination of microwave-assisted and ultrasonic-assisted extraction was the most effective method for extracting polysaccharides from *D. indusiata*, yielding 12.66%, compared to other methods, including ultrasonic extraction (11%), microwave extraction (10%), and hot water extraction (8.5%). A combination of pressure and hot water extraction was used to extract *β*-glucan from *Len*. *sajor-caju* under conditions of 140°C and 0.92 MPa for 40 min, resulting in a high yield of 3.20 g per 100 g of dry fruiting body (Sakdasri et al., 2022). Similarly, Tepsongkroh et al. (2023) applied high-pressure processing at 600 MPa for 10 min, combined with hot water extraction at 60°C for 2 h to extract polysaccharides from *V. volvacea*, resulting in a 12% increase in crude polysaccharide yield and a 20% increase in *β*-glucan yield. The combined extraction of polysaccharides from *G. lucidum* under optimal conditions (ultrasonic power of 240 W, enzyme concentration of 0.5 mg/mL, pH 7.9, solvent-to-material ratio of 50:1 mL/g, temperature of 55°C, and extraction time of 144 min) resulted in a yield of 3.72% of dried fruiting bodies (Hoa, 2018). The combination of enzyme-assisted extraction (1:1:1 ratio of papain, pectinase, and cellulase, a solvent-to-material ratio of 1:30, pH 5, a temperature of 48°C), and microwave-assisted extraction (440 W for 10 min) for the extraction of EnPs from *Le. edodes* resulted in a high yield of 9.79% of dried fruiting bodies (Yin et al., 2018). Recently, Yang et al. (2024d) found that the enzyme-assisted extraction technique, combined with ultrasonic-assisted extraction, effectively extracted polysaccharides from *F. velutipes* under optimal conditions (ultrasonic power of 150 W, snailase enzyme concentration of 1%, solvent-to-material ratio of 10:1 mL/g, temperature of 55°C, and extraction time of 90 min), resulting in a polysaccharide yield of 109.54 mg/g of dried fruiting bodies.

Thus, the selection of an extraction method of fungal polysaccharide must consider multiple factors, including the type of sample, extraction efficiency, cost, time, and environmental impact, in order to achieve the highest yield of fungal polysaccharides. Additionally, treatment temperature, solvent-to-sample ratio, and energy used in the extraction process all significantly affect polysaccharide yields. It is essential to optimize these variables to efficiently extract fungal polysaccharides and achieve high yields, along with the combination of extraction techniques.

## 6 Precipitation techniques for fungal polysaccharides

The precipitation of fungal polysaccharides is a process used to isolate these polysaccharides from a solution by adding a chemical agent, such as ethanol, propanol, or isopropyl alcohol, in an appropriate ratio. Ethanol is typically used to directly precipitate crude polysaccharides from the fungal extract solution (Moradi and Kalanpour, 2019; Miao et al., 2020). The use of ethanol for precipitation reduces the solubility and hydrophilic properties of polysaccharides in water (Monroy et al., 2016; Meng et al., 2023). Furthermore, precipitation at lower temperatures, such as incubation at 4°C, enhances polysaccharide precipitation (Wang et al., 2022b, 2023a, b). The precipitation of fungal polysaccharides using ethanol involves varying concentrations and volumes, depending on the type of crude polysaccharide and the extraction method employed. Previous studies reported that ethanol concentrations used for fungal polysaccharide precipitation typically range from 80% to absolute ethanol, with ethanol-to-sample ratios varying from 1:1 (*v*/*v*) to 1:5 (*v*/*v*), usually under cold conditions (4°C) and over an overnight period (Liu et al., 2018; Gao et al., 2022; Sakdasri et al., 2022; Liu et al., 2024a; Panya et al., 2024; Sahib et al., 2024). After precipitation, the polysaccharides can be separated by filtration or centrifugation and then dried by either conventional drying or lyophilization to obtain a crude solid form. The crude polysaccharides are usually kept dry to prevent moisture and microbial contamination and are used for further studies.

## 7 Purification techniques for fungal polysaccharides

For research and biological applications, it is essential to purify crude fungal polysaccharides in order to maintain the greatest biological properties and achieve high purity. Crude fungal polysaccharides contain a variety of contaminants, including pigments, proteins, monosaccharides, and other compounds, as a result of the limitations of extraction techniques (Wang et al., 2022b). Protein removal and decolorization of crude polysaccharides are commonly achieved using the sevage method and hydrogen peroxide treatment, respectively (Li et al., 2019b; Hu et al., 2022b). Generally, purification methods for polysaccharides include anion exchange chromatography and gel permeation chromatography. Following purification, the polysaccharides are concentrated, dialyzed, and freeze-dried. Finally, the polysaccharide and protein contents in the purified polysaccharides are quantified using the dinitrosalicylic acid, Folin-Ciocalteu, or phenol-sulfuric acid methods (Wood et al., 2012; Saravanakumar et al., 2021; Gao et al., 2022).

### 7.1 Anion exchange chromatography

Anion exchange chromatography is a crucial technique for purifying fungal polysaccharides, particularly those with a negative charge, such as acidic polysaccharides. This method relies on the principle of ion exchange, where the sample is passed through a column containing a medium capable of binding anions, such as DEAE-cellulose 52 or DEAE-Sephadex A-25, which have positively charged functional groups on their surfaces. When the polysaccharide is introduced into the column, negatively charged polysaccharides are adsorbed onto the medium via ion exchange. Subsequently, the bound polysaccharides are eluted from the column using a buffer solution with increasing salt concentration, which helps release the retained polysaccharides. This technique is well-suited for separating polysaccharides with varying structures and charges, enhancing extraction efficiency, and significantly reducing impurities in fungal polysaccharides (Ren et al., 2019; Wang et al., 2022b).

### 7.2 Gel permeation chromatography

Gel permeation chromatography is a widely used technique for purifying fungal polysaccharides, particularly for separating substances according to molecular size. The principle of gel permeation chromatography involves the use of a gel with a porous structure within the column, which facilitates the separation of sample molecules based on size. The polysaccharides are injected into a column packed with gel, such as Sephadex G-100 or Sephadex G-200. As the solution flows through the column, larger molecules are unable to penetrate the pores of the gel effectively, allowing them to move through the column more quickly and elute first. In contrast, smaller molecules can penetrate the pores, causing them to migrate more slowly and elute later. Gel permeation chromatography is particularly suitable for isolating polysaccharides with varying molecular weights, effectively reducing contaminants and resulting in purer, more homogeneous polysaccharides (Ren et al., 2019; Wang et al., 2022b).

## 8 Techniques for analyzing the structural and compositional characteristics of fungal polysaccharides

Fungal polysaccharides are complex compounds with diverse molecular weights, monosaccharide compositions, types of glycosidic bonds, and backbone structures, all of which vary significantly across species and growth conditions. These polysaccharides typically consist of monosaccharide units, such as glucose, fructose, mannose, and galactose, linked by glycosidic bonds, and may adopt either linear or branched structural configurations (Sun et al., 2022b; Yang et al., 2022a). Advanced analytical techniques, including gel permeation chromatography (GPC), high-pressure gel permeation chromatography (HPGPC), gas chromatography-mass spectrometry (GC-MS), high-performance liquid chromatography (HPLC), Fourier transform infrared (FT-IR) spectroscopy, nuclear magnetic resonance (NMR), and scanning electron microscopy (SEM), are employed to investigate the specific structural characteristics of fungal polysaccharides (Wang et al., 2022c; Zhao et al., 2023a). Thus, understanding these structural and functional properties is critical for advancing their potential applications and can lead to innovative product development that maximizes the benefits of fungal polysaccharides. Examples of monosaccharide composition, structure, and characterization methods of fungal polysaccharides are compiled and presented in **Table 5**.

**Table 5:** Examples of the monosaccharide composition of fungal polysaccharides, structure, and methods of characterization.

Fungal Species	Type of Polysaccrahide	Monosaccharide Composition and Structure	Methods of Characterization	Reference
*Agrocybe cylindracea*	EnP	Arabinose, galactose, glucose, glucuronic acid, galacturonic acid, mannose, ribose, rhamnose, and xylose	GC-MS and FT-IR spectroscopy	Sun et al. (2022a)
*Aspergillus terreus*	ExP	Glucosamine, fucose, glucose and galactose with (1→3) and (1→6) of glycosidic linkages	HPLC, FT-IR, and NMR spectroscopy	Amer et al. (2020)
*Auricularia auricula*	EnP	Fucose, galactose, glucose, mannose, rhamnose, and xylose with molecular weight of 173 kDa	GPC, HPLC, and FT-IR spectroscopy	Xiang et al. (2021)
*Auricularia cornea*	EnP	Fucose, galactose, glucose, glucuronic acid, mannitol, rhamnose, and xylose	GC-MS	Fu et al. (2022)
*Auricularia polytricha*	EnP	Fucose, galactose, glucose, mannose, rhamnose, and xylose with molecular weight of 17.1 kDa	GPC, HPLC, and FT-IR spectroscopy	Xiang et al. (2021)
*Cordyceps militaris*	EnP	Backbone of (1→4)-*β*-D-Gl*cp* and (1→2)-*α*-D-Ma*np* glycosyls and with a molecular weight of 700 kDa.	HPGPC, HPLC, FT-IR, and NMR spectroscopy	Yang et al. (2021b)
*Craterellus tubaeformis*	EnP	Arabinose, fucose, galactose, glucose, mannose, rhamnose, and xylose with *α*- and *β*-D-mannopyranose, D-glucopyranose, D-galactopyranose, *α*-l-arabinofuranose, and *α*-l-fucopyranose with molecular weight of 2.86 and 783 kDa	GC-MS, FT-IR, and NMR spectroscopy	Deveci et al. (2024)
*Curvularia lunata*	ExP	Galactose, glucose, and mannose with (1→3)-*β*-glycosidic linkages	HPLC, FT-IR, and NMR spectroscopy	Jayus et al. (2021)
*Dictyophora indusiata*	EnP	Galactose, glucose, and mannose.	HPLC	Kanwal et al. (2020)
*Flammulina velutipes*	EnP	Fucose, galactose, glucose and mannose with linkages of →4)-*α*-D-Gal*p*(1→, →4,6)-*α*-D-Gal*p*(1→, →2)-*α*-L-Fuc*p*-(1→, →3,6)-*α*-D-Gal*p*-(1→, *α*-D-Man*p*-(1→, and →3)-*β*-D-Glcp-(1→, and molecular weight of 15 kDa	GPC, HPLC, FT-IR, and NMR spectroscopy	Wang et al. (2018)
*Fusarium nygamai*	ExP	Fructose and glucose. Composing of glycosidic bond	HPLC and FT-IR spectroscopy	El-Mahdy et al. (2023)
*Ganoderma atrum*	EnP	Arabinose glucose, galactose, mannose, and rhamnose with molecular weight of 1,013 kDa	GPC, HPLC, and FT-IR spectroscopy	Wu et al. (2022a)
*Ganoderma cantharelloideum*	ExP	Rhamnose, glucose, galactose, mannose, and xylose with molecular weight of 627.72 and 74.56 kDa. Composing of *α*- and *β*-glycosidic bonds.	GPC, GC-MS, and FT-IR spectroscopy	Long et al. (2021)
*Grifola frondosa*	EnP	Fucose, galactose and glucose, with linkages of →4)-*α*-D-Gl*cp*-(1→, *β*-D-Gl*cp*-(1→ and →4,6)-*β*-D-Gl*cp*-(1→ with molecular weight of 5,570 kDa	GPC, GC-MS, HPLC, FT-IR, and NMR spectroscopy	Jiang et al. (2022)
*Hygrophorus pudorinus*	EnP	Arabinose, fucose, galactose, glucose, and mannose with *α*- and *β*-D-mannopyranose, D-glucopyranose, D-galactopyranose, *α*-l-arabinofuranose, and *α*-l-fucopyranose with molecular weight of 3.09 and 14.6 kDa	GC-MS, FT-IR, and NMR spectroscopy	Deveci et al. (2024)
*Inonotus obliquus*	EnP	Arabinose, fucose, galactose, glucose, glucuronic acid, galacturonic acid, mannose, rhamnose, and xylose with molecular weight of 373 kDa.	HPLC, FT-IR, and HPGPC	Su et al. (2022)
*Lentinula edodes*	EnP	Mannose, glucosamine, glucuronic acid, glucose, galactose and fucose. Composing of (1→6)-*β*-D-glucans with minor *β*-(1→3) glucosidic side chains	HPGPC, HPLC, FT-IR, and NMR spectroscopy	Li et al. (2019a)
	ExP (Lentinan)	Backbone consists of β-(1,3)-glucan with *β*-(1,6) branching	GPC, FT-IR, and NMR spectroscopy	Sahib et al. (2024)
*Lyophyllum decastes*	EnP	Mannose, glucose, galactose, and fucose with linkages of 1,3-Fuc*p*, T-Gal*p*, 1,4-Glu*p*, 1,6-Glu*p*, 1,6-Gal*p*, and 1,2,6-Man*p*	HPLC, GC-MS, SEM, FT-IR, and NMR spectroscopy	Wang et al. (2022b)
*Macrolepiota procera*	EnP	Arabinose, fucose, galactose, glucose, and mannose with *α*- and *β*-D-mannopyranose, D-glucopyranose, D-galactopyranose, *α*-l-arabinofuranose, and *α*-l-fucopyranose with molecular weight of 3.21 and 12.1 kDa	GC-MS, FT-IR, and NMR spectroscopy	Deveci et al. (2024)
*Phellinus linteus*	EnP	Arabinose, fucose, galactose, glucose, mannose, and xylose with linkages of (1→4)-*α*-D-glucose (1→6), *α*-(1→3)-D-glucose, and *α*-(1→6)-D-glucose with molecular weight of 250,000 and 28,000 kDa.	GC-MS, FT-IR, and NMR spectroscopy	Mei et al. (2015)
*Pleurotus eryngii*	EnP	Rhamnose, arabinose, xylose, mannose, glucose, and galactose. Composing of *α*- and *β*-glycosidic bonds.	HPGPC, HPLC, FT-IR, and NMR spectroscopy	Gong et al. (2022)
*Pleurotus geesterani*	ExP	Arabinose, galactose, glucose, mannose, rhamnose, and xylose. Composing of *α*-glycosidic bond	GC-MS, FT-IR, NMR spectroscopy, and SEM	Song et al. (2018)
*Pleurotus pulmonarius*	ExP	Mannose, glucose, and galactose. Composing of (1 → 3), (1 → 6)-linked *β*-D-glucans	GC-MS, FT-IR, and NMR spectroscopy	Smiderle et al. (2012)

### 8.1 GPC and HPGPC

GPC and HPGPC have been used to separate and analyze molecular sizes based on the principle of size exclusion. Larger molecules elute earlier from the column, while smaller molecules, which can penetrate the pores of the gel more effectively, elute later. GPC is particularly suited for determining average molecular weights and molecular weight distributions in polymers, proteins, and polysaccharides. In contrast, HPGPC is an advanced version of GPC, utilizing high-pressure systems and columns with smaller particle sizes to enhance resolution, speed, and accuracy (Ren et al., 2019). Both GPC and HPGPC are techniques that have been used to analyze polysaccharides from fungi with diverse molecular weights. For example, previous studies on the molecular weight of EnPs and ExPs from *Co. militaris* found that the HPGPC separates low-molecular-weight polysaccharides more effectively than the GPC, which can only obtain molecular weights lower than 100 kDa (Wang et al., 2015a; Huang et al., 2018; Kang et al., 2022; Liu et al., 2024; Wang et al., 2024). Similarly, studies on the molecular weight of EnPs and ExPs from *Le. edodes* found that HPGPC is more efficient than GPC at separating low-molecular-weight polysaccharides (Li et al., 2018a; Ren et al., 2018; Zhang et al., 2022a). Moreover, molecular weight analysis of both EnPs and ExPs from *He. erinaceus* revealed that HPGPC is more effective at separating low-molecular-weight polysaccharides below 10 kDa compared to GPC (Wang et al., 2015b; Wu et al., 2017, 2018). Based on the previous studies mentioned, HPGPC demonstrates the capability to separate smaller fungal polysaccharides with higher effectiveness than GPC.

### 8.2 GC-MS

GC is a technique used to separate components of a mixture in the gas phase based on differences in solubility and adsorption properties of each substance. This technique involves of two phases: a mobile phase, which is a carrier gas, and a stationary phase, which can be a liquid, solid, or semi-solid material contained within a column. Substances with different chemical characteristics move through the column at varying speeds, depending on their properties, such as solubility or interaction with the stationary phase. This technique allows for detailed analysis of monosaccharide composition in fungal polysaccharides with exceptional accuracy (Millette et al., 2023). For example, GC analysis revealed that arabinose, galactose, glucose, mannose, and xylose are the monosaccharides present in the composition of EnPs extracted from *Co. militaris* (Luo et al., 2017; He et al., 2020a; Liu et al., 2024c). Studies on the monosaccharide profile of EnPs from *Le. edodes* revealed the composition of rhamnose, arabinose, xylose, mannose, galactose, and glucose through GC analysis (Li et al., 2018a; Zhang et al., 2022a). The monosaccharide composition of the EnPs from *G. resinaceum* consists of glucose, galactose, mannose, fucose, and xylose, as determined by GC analysis (Bleha et al., 2022). Additionally, GC techniques have also been employed to determine the monosaccharide composition in ExPs. For instance, in the study by Vanin et al. (2023), the monosaccharide composition of ExPs from *Sch. commune* was found to be primarily composed of glucose, followed by xylose, mannose, galactose, and arabinose in smaller amounts. Furthermore, the study by Ren et al. (2018) investigating lentinan, an ExPs from *Le. edodes*, using GC analysis revealed that its monosaccharide composition consists of glucose, mannose, and galactose.

### 8.3 HPLC

HPLC is used to analyze and separate the components of fungal polysaccharides through precise chromatographic techniques. Prepared samples are introduced into an HPLC column packed with specialized separation media. As the solution flows through the column under high pressure, molecules interact with the column material according to their chemical characteristics and molecular size, enabling efficient separation. Upon elution, a detector captures the concentration of each component, yielding data for detailed analysis of monosaccharide composition, concentration quantification, and the assessment of polysaccharide degradation across various processes (Ingale et al., 2023). Using HPLC, Liu et al. (2018) examined the monosaccharide composition of EnPs isolated from *Pax. involutus* and found that glucose was the main component, followed by galactose, mannose, and fucose. Wang et al. (2024) investigated the monosaccharide composition of four EnPs isolated from *Co. militaris* using HPLC, finding that glucose and galactose were present, with glucose being the predominant component. In the study by Chen et al. (2020), HPLC was used to analyze the monosaccharide composition of four different EnPs extracted from *Sch. commune* obtained through various extraction methods. The results revealed that mannose, ribose, rhamnose, glucuronic acid, galacturonic acid, glucose, galactose, xylose, arabinose, and fucose were present in varying ratios, with glucuronic acid being the main component. Additionally, Ayimbila et al. (2021) analyzed the monosaccharide composition of EnPs extracted from *Len. squarrosulus* using HPLC, revealing that the polysaccharides were primarily composed of glucose, followed by galactose, mannose, and fucose. The HPLC technique has also been employed to analyze the monosaccharide composition of ExPs. For instance, a study reported that lentinan, an ExP derived from *Le. edodes*, predominantly consists of glucose as the main monosaccharide, with minor amounts of galactose and mannose detected (Xu et al., 2023). Additionally, the study by Kang et al. (2022) also revealed that the monosaccharide composition of ExPs from *Co. militaris* consists primarily of glucose, with smaller amounts of galactose and mannose. Comparative studies using HPLC and GC to analyze the monosaccharide composition of fungal polysaccharides have shown no significant differences, with the primary components being glucose, mannose, and galactose. However, arabinose, fucose, ribose, rhamnose, and xylose can also be found in some fungal polysaccharides.

### 8.4 FT-IR spectroscopy

FT-IR spectroscopy is an essential analytical method for studying fungal polysaccharides. This technique functions by inducing vibrational modes in molecular bonds via infrared radiation, which is then absorbed by the sample. The resulting absorption data is processed into a spectrum, presenting the frequency and absorption intensity relationship. This enables detailed structural analysis and functional group identification, such as glucose and fructose residues, allowing for precise assessments of sample purity and detection of potential impurities within the polysaccharide material (Hong et al., 2021). The study by He et al. (2020a) analyzed EnPs from *Co. militaris* using FT-IR spectroscopy, revealing a strong and broad absorption peak at 3392.8 cm^−1^, attributed to O-H stretching vibrations of hydroxyl groups due to intramolecular or intermolecular interactions, along with a weaker absorption band at 2930.6 cm^−1^, corresponding to C-H stretching vibrations in -CH_3_ or -CH_2_ groups. Similar to the study by Wang et al. (2024), which analyzed EnPs from *Co. militaris* to examine organic functional groups, the broad absorption peak at 3400 cm^−1^ was attributed to O-H stretching vibrations from intermolecular or intramolecular interactions within the polysaccharide, while the weaker absorption band at 2925 to 2931 cm^−1^ corresponded to asymmetric C-H stretching vibrations in sugar groups. In the study by Zhang et al. (2022a), the functional groups in *Le. edodes* EnPs were observed, with the strong absorption peak near 3300 cm^-1^ attributed to O-H stretching vibrations, and the signal at 2922 cm^-1^ resulting from C-H stretching vibrations; strong absorptions around 1600 cm^-1^ indicated C=O stretching, while the absorption near 1400 cm^-1^ was due to C-H deformation vibrations; C-O-C group signals between 1000–1100 cm^-1^ confirmed pyranose ring linkages, and the weaker signal at 890 cm^-1^ reflected β-glycosidic bonds. Ascencio et al. (2021) investigated the functional groups of lasiodiplodan from *L. theobromae* and revealed distinct spectral characteristics, including broadband with high intensity at 3274 cm^-1^ corresponding to the stretching vibration of R-OH groups, a band at 1650 cm^-1^ associated with the glucose ring structure, symmetric vibrations of the C-O-C bond characteristic of carbohydrates exhibiting absorption at 1075 cm^-1^, and a low-intensity band at 890 cm^-1^ indicative of glycosidic bonds representing the β-configuration arrangement. Based on the previous research examples provided, it can be observed that the strong and broad absorption peak is commonly attributed to the O-H hydroxyl group, which is frequently found in fungal polysaccharides. However, other functional groups, including C-H, C=O, and C-O-C, also appear, emphasizing the chemical diversity in fungal polysaccharides and reflecting the unique properties and structures found across different fungal species (He et al., 2020a; Zhang et al., 2022a).

### 8.5 NMR spectroscopy

NMR spectroscopy is a valuable tool for analyzing the structural properties of fungal polysaccharides by exploiting the behavior of atomic nuclei in a powerful magnetic field. Nuclei such as hydrogen (¹H) and carbon (¹³C) resonate at characteristic frequencies, absorbing energy in ways that reveal precise structural details. This technology allows for accurate determination of polysaccharide structure, including the sequence of monosaccharide units and the types of glycosidic linkages present (Yao et al., 2021). NMR spectroscopy was used to investigate the glycosidic bond structure of EnPs derived from *Mor. sextelata*, revealing that the EnPs contain glycosidic linkages similar to (1→4)-linked-*α*-Glc*p* suggesting a specific type of linkage in its structure Li et al. (2022). In the study by Tepsongkroh et al. (2023), NMR spectroscopy was used to examine the glycosidic bonds of polysaccharides from *V. volvacea*, revealing that the polysaccharide consists of (1→3)-linked-*β*-D-Glc*p* glycosidic linkages, forming a *β*-glucan (1,3/1,6) structure. Some studies have revealed the complexity of glycosidic bond structures, such as a research by Ayimbila et al. (2021), where NMR spectroscopy was used to analyze the glycosidic bond structure of EnPs derived from *Len. squarrosulus*. The analysis identified a complex structure composed of (1→4,6)-linked-*β*-D-Glc*p*, (1→4)-linked-*β*-D-Glc*p*, (1→6)-linked-*β*-D-Glc*p*, (1→4)-linked-*β*-D-Man*p*, *α*-L-fucose, and (1→6)-linked-*α*-galactosyl, highlighting the structural diversity of these EnPs. Zhang et al. (2023) found that glycosidic bond structure of EnPs derived from *D. indusiata* revealed a complex structure consisting of (1→6)-linked-*β*-Glc*p*, (1→2,6)-linked-*α*-Glc*p*, (1→3)-linked-*α*-Man*p*, (1→6)-linked-*β*-Man*p*, and (1→6)-linked-*β*-Gal*p*. Additionally, lentinan from *Le. edodes*, analyzed for glycosidic bond structure using NMR spectroscopy, revealed that its backbone consists of *β*-(1,3)-glucan with *β*-(1,6) branching (Sahib et al., 2024). The NMR spectroscopy technique aids in identifying the types of glycosidic linkages, such as *β*-D-glucan and *α*-D-glucan, by detecting specific signals in the spectra. The most commonly observed glycosidic linkages include (1→3)-linked-*β*-Glc*p*, (1→6)-linked-*β*-Glc*p*, (1→3)-linked-α-Glc*p*, and (1→6)-linked-*α*-Glc*p* (Ayimbila et al., 2021; Gao et al., 2022; Li et al., 2022; Tepsongkroh et al., 2023; Zhang et al., 2023). This technique is crucial for understanding the types of glycosidic bonds and the backbone structures of fungal polysaccharides, which in turn influence the potential applications.

### 8.6 SEM

SEM is employed to examine the physical characteristics of fungal polysaccharides by using a high-energy electron beam to capture highly detailed surface images at the nanometer scale. This process begins with sample preparation, which typically includes applying a metallic coating to enhance conductivity. When the electron beam interacts with the sample surface, it generates scattered electrons and photons that are detected to produce a three-dimensional representation. SEM allows for precise analysis of structural features, including particle size, surface morphology, and distribution within the polysaccharide matrix (Fellak et al., 2022). In the study by Li et al. (2022), SEM analysis of EnPs from *Mor. sextelata* revealed the sponge-like porous structure with a smooth surface. Similarly, the study by Qiu et al. (2024) reported that the surface morphology of EnPs extracted from *Pl. eryngii* exhibited a sponge-like porous structure. A comparable sponge-like porous structure was also observed in pullulan, an exopolysaccharide extracted from *Au. pullulans* (Maia et al., 2023). Additionally, different extraction methods influence the surface morphology and structure of fungal polysaccharides. Each method affects the integrity and arrangement of molecules within the polysaccharide structure (Deng et al., 2015; Chemat et al., 2017). In the study by Zhang et al. (2022a), SEM was used to examine the surface morphology of EnPs from *Le*. *edodes* extracted using different methods. The polysaccharide extracted via subcritical water extraction with a deep eutectic solvent showed a loose, rough, and porous structure. The polysaccharide from subcritical water extraction alone exhibited a leaf-like surface with uneven distribution but a smooth and fine texture. The polysaccharide extracted by hot water extraction displayed a sheet-like structure with irregular aggregation. Moreover, in the study by Rosyida et al. (2024), EnPs from *Pl. ostreatus* were extracted using various techniques. The results revealed that polysaccharides extracted through kinetic-assisted hot extraction exhibited fewer pores and a smoother surface. In contrast, those extracted by microwave and ultrasound-assisted extraction displayed puffed structures, numerous open cavities, and collapsed surfaces. Furthermore, SEM has been employed to examine the surface morphology of exopolysaccharides, such as pullulan derived from *Au. pullulans*, which exhibits a smooth surface structure (Tagne et al., 2024), and lentinan from *Le. edodes*, characterized by a rough surface and irregular pores resembling a honeycomb-like porous structure (Xu et al., 2023).

## 9 Properties of fungal polysaccharides

Fungal polysaccharides have garnered significant interest in recent years due to their diverse and potent properties. These polysaccharides exhibit a wide range of beneficial effects, including antidiabetic, antioxidant, antiviral, antilipidemic, antitumor, and immunomodulating properties. An example of the biological properties of fungal polysaccharides is shown in **Table 6**.

**Table 6:** Examples of the biological properties of fungal polysaccharides.

Biological activity	Fungal species	Type	Experimental model	Effect	Reference
Antidiabetic property	*Auricularia auricula*	EnP	Streptozotocin-induced diabetic mice	After 4 weeks of administration at a dose of 100 mg/kg body weight, EnP reduced body weight and fasting blood glucose levels while increasing serum insulin in diabetic mice.	Xiang et al. (2021)
	*Auricularia polytricha*	EnP	Streptozotocin-induced diabetic mice	After 4 weeks of administration at a dose of 100 mg/kg body weight, EnP reduced body weight and fasting blood glucose levels while increasing serum insulin in diabetic mice.	Xiang et al. (2021)
	*Cordyceps taii*	EnP	Streptozotocin-induced diabetic mice	Oral administration of EnP at a dose of 100 mg/kg body weight for 28 days resulted in a 36.13% reduction in body weight, a 32.47% decrease in fasting serum insulin levels, a 56.79% reduction in fasting blood glucose, while also improving and repairing impaired pancreatic islet *β*-cells.	Liu et al. (2019)
	*Dictyophora indusiata*	EnP	High-fat emulsion-induced mice	EnP at a dose of 400 mg/kg body weight can detect and inhibit the increase in blood sugar levels in mice with hyperlipidemia.	Wang et al. (2019b)
	*Grifola frondosa*	EnP	Streptozotocin-induced diabetic mice	Oral administration of EnP at a dose of 75 mg/kg body weight reduced fasting blood glucose levels, improved oral glucose tolerance, alleviated insulin resistance, and mitigated hepatic insulin resistance by modulating the IRS1/PI3K and JNK signaling pathways in type 2 diabetic mice.	Chen et al. (2019)
		EnP	HepG2 cell lines and type 2 diabetic mice	EnP enhanced glucose uptake and alleviated insulin resistance in HepG2 cells, while improving blood glucose levels and glucose tolerance in type 2 diabetic mice.	Chen et al. (2018)
	*Pleurotus eryngii*	ExP	Streptozotocin-induced diabetic mice	Oral administration of ExP at 600 mg/kg body weight reduced blood glucose levels by 49.15% in diabetic mice, while *in vitro*, it inhibited *α*-amylase and *α*-glucosidase activities by 62.36% and 42.38%, respectively.	Zhang et al. (2018a)
	*Pleurotus geesterani*	ExP	Streptozotocin-induced diabetic mice	After 3 weeks of administration at a dose of 200 mg/kg body weight, ExP reduced plasma glucose levels by 17.1% in diabetic mice, repaired streptozotocin-induced pancreatic *β*-cell damage, and functioned as an insulin-like factor to stimulate insulin synthesis.	Duobin et al. (2013)
	*Pleurotus ostreatus*	EnP	High-fat-high-cholesterol emulsion-induced liver injured mice	Oral administration of EnP at a dosage of 400 mg/kg reduced blood glucose levels by 32.76% to normal at 120 min, compared to the control group.	Dong et al. (2019)
	*Suillellus luridus*	EnP	Streptozotocin-induced diabetic mice	EnP administered at a dose of 150 mg/kg body weight, demonstrated significant antidiabetic effects by improving weight loss and increasing serum insulin levels in type 2 diabetic mice.	Zhang et al. (2018b)
Antilipidemic property	*Agaricus blazei*	EnP	Oleic acid-induced HepG2 cells and high-fat diet-induced rat	EnP decreased TC and TG levels in HepG2 cells, reduced serum levels of TC, TG, and LDL-C, and increased serum HDL-C levels in rats.	Li et al. (2020b)
	*Auricularia auricular*	EnP	Cholesterol-enriched diet-induced mice	Oral administration of EnP at a dose of 100 mg/kg body weight for 2 weeks reduced serum TC and TG levels by 46.6% and 46.4%, respectively.	Zeng et al. (2013)
	*Botryosphaeria rhodina*	ExP (Botryosphaeran)	Streptozotocin-induced diabetic mice	Oral administration of botryosphaeran at a dose of 12 mg/kg body weight per day for 15 days resulted in reductions in plasma TC and LDL-C levels by 18% and 27%, respectively, in hyperlipidemic rats.	Miranda-Nantes et al. (2011)
	*Cordyceps militaris*	EnP	High-fat emulsion-induced mice	Oral administration of EnP at a dose of 400 mg/kg body weight reduced serum TC, TG, HDL-C, LDL-C, and VLDL-C to 2.26 mmol/L, 0.68 mmol/L, 1.93 mmol/L, 0.81 mmol/L, 0.29 mmol/L, respectively, compared to the control group.	Wang et al. (2015b)
	*Cordyceps taii*	EnP	Streptozotocin-induced diabetic mice	Oral administration of EnP at a dose of 100 mg/kg body weight for 28 days reduced TC, TG, and LDL-C levels by 13.84%, 31.87%, and 36.61%, respectively, and increased HDL-C levels by 28.60% compared to the control group.	Liu et al. (2019)
	*Dictyophora indusiata*	EnP	High-fat emulsion-induced mice	Oral administration of EnP at a dose of 400 mg/kg body weight reduced serum TC, TG, and LDL-C levels to 2.67 mmol/L, 1.65 mmol/L, and 1.09 mmol/L, respectively, compared to the control group.	Wang et al. (2019b)
	*Ganoderma applanatum*	EnP	Maize, cassava, palm oil, and sugar (MACAPOS-2)-induced obese rats	Oral administration of EnP at a dose of 150 mg/kg body weight lowered serum TC, TG, and LDL-C levels by 31.64%, 9.56%, and 43.52%, respectively, while reducing hepatic TC and TG levels by 41.05% and 38.28%, respectively.	Mfopa et al. (2021)
	*Helvella leucopus*	EnP	High-fat diet-induced mice	Oral administration of EnP at a dose of 60 mg/kg body weight for 6 weeks reduced TC, TG, and LDL-C levels, increased HDL-C levels, and regulated the expression of genes associated with hepatic lipid metabolism.	Ge et al. (2022)
	*Inonotus obliquus*	EnP	Oleic acid-induced HepG2 cells and high-fat diet-induced mice	EnP at a concentration of 60 mg/L decreased TC, TG, and LDL-C levels while increasing HDL-C content in HepG2 cells. In mice, oral administration of EnPS at a dose of 60 mg/L for 10 weeks reduced body weight and lowered TG and LDL-C levels by 24.8% and 30.1%, respectively, compared to the control group.	Yang et al. (2021a)
	*Pleurotus ferulae*	ExP	Streptozotocin-induced diabetic mice	Administration of ExP at a dose of 250 mg/kg body weight effectively lowered serum TG and LDL-C levels by 45.4% and 26.9%, respectively.	Wang et al. (2014b)
	*Pleurotus geesterani*	ExP	Streptozotocin-induced diabetic mice	Administration of ExP at a dose of 200 mg/kg body weight reduced plasma glucose, TC, and TG levels by 17.1%, 18.8%, and 12.0%, respectively, in mice.	Duobin et al. (2013)
	*Pleurotus ostreatus*	EnP	High-fat-high-cholesterol emulsion-induced liver injured mice	Oral administration of EnP at a dose of 400 mg/kg body weight decreased serum levels of LDL-C, TC, TG, alanine transaminase (ALT), aspartate transaminase (AST), alkaline phosphatase (ALP) while increasing HDL-C levels.	Dong et al. (2019)
Antimicrobial property	*Ganoderma applanatum*	ExP	Agar well diffusion method	ExP exhibited antibacterial activity against *Staphylococcus aureus*, demonstrating an inhibition zone of 17.9 mm and a minimum inhibitory concentration (MIC) of 1 mg/mL.	Osińska-Jaroszuk et al. (2014)
	*Ganoderma lucidum*	EnP	Microdilution method	EnP demonstrated antibacterial activity against *Staphylococcus epidermidis*, *Staphylococcus aureus*, *Bacillus subtilis*, *Micrococcus luteus*, and *Escherichia coli*, with MIC ranging from 0.63 to 1.25 mg/mL.	Skalicka-Woźniak et al. (2012)
		ExP	Agar well diffusion method	ExP demonstrated antibacterial activity against a diverse range of microorganisms, including *Escherichia coli*, *Staphylococcus aureus*, *Proteus* sp., *Bacillus subtilis*, *Pseudomonas aeruginosa*, *Klebsiella* sp., and *Bacillus cereus*.	Mahendran et al. (2013)
		EnP	Kirby-Bauer disc diffusion method	At a concentration of 500 mg/mL, EnP exhibited antimicrobial activity against a broad spectrum of microorganisms, including *Escherichia coli*, *Listeria monocytogenes*, *Shigella sonnei*, *Pseudomonas aeruginosa*, *S. enteritidis*, *Salmonella* spp., *Staphylococcus aureus*, *Staphylococcus epidermidis*, and methicillin-susceptible *Staphylococcus aureus*.	Wan-Mohtar et al. (2016)
	*Lentinus squarrosulus*	EnP	Agar well diffusion method	EnP demonstrated antibacterial activity with high sensitivity against *Salmonella enteritidis*, *Staphylococcus aureus*, and *Shigella dysenteriae* (MIC: 0.08 mg/mL) and lower sensitivity against *Escherichia coli*, *Salmonella typhimurium*, and *Salmonella gallinarum* (MIC: 0.63, 0.31, and 0.16 mg/mL, respectively).	Ayimbila et al. (2021)
Antioxidant property	*Cordyceps sinensis*	ExP	TEAC and FRAP scavenging activity assay	The ExP exhibited antioxidant activity, with TEAC values ranging from 35 to 40 µmol Trolox/g and FRAP values ranging from 50 to 52 µmol Fe (II)/g.	Leung et al. (2009)
		ExP	TEAC and hydroxyl scavenging activity assay	ExP demonstrated the highest antioxidant activity, with an EC_50_ value of approximately 0.13 mg/mL for scavenging hydroxyl radicals and a TEAC value of 66.5 μmol Trolox/g.	Yan et al. (2012)
	*Ganoderma lingzhi*	ExP	ABTS, DPPH, hydroxyl, and superoxide radical scavenging activity assay	ExP demonstrated antioxidant activities against ABTS, DPPH, hydroxyl, and superoxide radicals, with inhibition rates of 90.47%, 75.00%, 81.37%, and 61.26%, respectively.	Si et al. (2018)
	*Hericium coralloides*	ExP	DPPH radical scavenging activity assay	ExP exhibited DPPH radical scavenging activity, with an EC50 value of 6.59 mg/mL.	Tabibzadeh et al. (2022)
	*Lentinula edodes*	ExP (Lentinan)	DPPH radical scavenging activity assay	Lentinan exhibited strong DPPH radical scavenging activity in the group treated with water-extracted xylosma sawdust.	Lu et al. (2022)
	*Lentinus squarrosulus*	EnP	ABTS and DPPH radical scavenging activity assay	EnP demonstrated strong antioxidant activity, with IC_50_ values of 0.89 mg/mL for DPPH and 2.10 mg/mL for ABTS assays.	Ayimbila et al. (2021)
	*Paxillus involutus*	EnP	ABTS, DPPH, hydroxyl, and superoxide radical scavenging activity assay	EnP exhibited antioxidant activity, with IC_50_ values of 0.10 mg/mL for hydroxyl radical, 0.92 mg/mL for ABTS radical, 0.38 mg/mL for DPPH radical, and 94.94% for superoxide radical.	Liu et al. (2018)
	*Russula senecis*	EnP	ABTS, DPPH, hydroxyl, and superoxide radical scavenging activity assay	The reduction power of EnPS exhibited EC_50_ values ranging from 257 to 4068 µg/ml.	Khatua and Acharya (2017)
	*Schizophyllum commune*	ExP (Schizophyllan)	ABTS, FRAP, ORAC, and hydroxyl radical scavenging activity assay	Schizophyllan effectively inhibited free radicals, demonstrating strong antioxidant activities against ABTS, FRAP, ORAC, and hydroxyl radicals.	Deng et al. (2021)
	*Tricholoma mongolicum*	EnP	ABTS, DPPH, and hydroxyl scavenging activity assay	The reduction power of EnP exhibited IC_50_ values ranging from 0.80 to 1.27 mg/ml.	Zhang et al. (2022b)
Anticancer property	*Aureobasidium pullulans*	ExP (Pullulan)	HeLa cell lines	Nanoparticles formulated with pullulan and encapsulated with anticancer drugs demonstrate enhanced potency in eliminating drug-resistant HeLa cells compared to nanoparticles containing the drug alone.	Wu et al. (2022b)
	*Botryosphaeria rhodina*	ExP (Botryosphaeran)	Obese tumor-induced rats	Botryosphaeran suppresses tumor progression, alleviates body weight loss and cachexia, reduces mesenteric fat and insulin resistance, and improves macrocytic anemia	Geraldelli et al. (2020)
	*Fomitiporia chilensis*	EnP	HCT-116 colorectal cancer cell lines	Treatment of HTC-116 cells with EnP at a concentration of 2.0 mg/mL induced an increase in the G0/G1 cell cycle phase and elevated the apoptotic cell percentage to 16.6%.	Abdala-Díaz et al. (2024)
	*Ganoderma lucidum*	EnP	Human gastric cancer AGS cell lines	EnP from sporoderm-removed spores of *G. lucidum* inhibited cell viability at 2.60 mg/mL after 72 hours and modulated key apoptotic and autophagy markers, including downregulation of B-cell lymphoma 2	Zhong et al. (2021)
		EnP	4T1-breast cancer xenograft mice	Administration of EnP at a dose of 400 mg/kg body weight reduced tumor weight from 512 mg to 387 mg and increased both the cytotoxic T cell population and the cytotoxic T cell-to-helper T cell ratio in the peripheral blood of tumor-bearing mice.	Su et al. (2018)
		EnP	Mouse sarcoma S180-bearing mice	Administration of EnP at a dose of 30 mg/kg body weight reduced the proliferation of S180 cells, decreasing tumor weight from 1.45 g to 0.82 g.	Fu et al. (2019)
	*Grifola frondosa*	EnP	Heps tumor-bearing mice	Administration of EnP at a dose of 54 mg/kg body weight reduced tumor weight from 1.46 g to 0.52 g, achieving an inhibition rate of 64.38%.	Mao et al. (2016)
	*Inonotus obliquus*	EnP	Human T lymphadenoma jurkat tumor-bearing mice	Administration of EnP at a dose of 80 mg/kg body weight reduced tumor weight from 8.66 g to 2.66 g, achieving an inhibition rate of 69.28%.	Chen et al. (2015)
	*Lasiodiplodia theobromae*	ExP (Lasiodiplodan)	MCF-7 breast cancer cell lines	Lasiodiplodan demonstrated an inhibitory effect on the proliferation of MCF-7 breast cancer cells, while its sulfonated derivative exhibited anticoagulant and antithrombotic activities comparable to those of heparin.	Alves da Cunha et al. (2012)
	*Lentinula edodes*	EnP	HCT-116 colorectal cancer and HeLa cervical cancer cell lines	EnP at a 1.79 mg/mL concentration inhibited the proliferation of HCT-116 and HeLa cells. The proliferation ratios were 28.9 to 36.4% for HCT-116 cells and 26.7 to 32.5% for HeLa cells when treated with EnPS.	Zhao et al. (2016b)
		ExP (Lentinan)	78 patients with metastatic or recurrent gastric cancer	Lentinan, when used in conjunction with other chemotherapeutic agents, has demonstrated efficacy in alleviating common side effects of chemotherapy, including nausea, discomfort, hair loss, and compromised immune function.	Ina et al. (2011)
	*Lentinus velutinus*	EnP	HeLa cervical cancer and HepG2 human hepatoblastoma cell lines	At a concentration of 2000 µg/mL, EnP inhibited the proliferation of HeLa and HepG2 cells by 22.44% and 21.92%, respectively, after 24 hours of treatment and by 49.97% and 51.83%, respectively, after 48 hours of treatment.	Udchumpisai and Bangyeekhun (2020)
	*Phellinus pullus*	EnP	Mouse sarcoma S180 cell lines and tumor-bearing mice	Administration of EnP at a dose of 6 g/kg body weight inhibited the proliferation of S180 cells and suppressed tumor growth in S180-transplanted mice, achieving a maximum antitumor rate of 85.47%.	Yang et al. (2016)
	*Schizophyllum commune*	ExP (Schizophyllan)	Dimethylbenz(α)anthracene (DMBA)-induced carcinomas in mice	Schizophyllan exhibits the potential to inhibit estrogen receptor-positive breast cancer in a manner comparable to tamoxifen while mitigating liver damage associated with tamoxifen treatment in experimental mice.	Mansour et al. (2012)
Antiviral property	*Agaricus brasiliensis*	EnP	Bovine herpesvirus 1 (BoHV-1)-infected HEp-2 cell lines	The polysaccharide-peptide demonstrated antiviral inhibition of 67.9%, while *β*-glucan exhibited a high inhibition of virus replication, with 83.2% inhibition in the plaque assay and 63.8% inhibition in the immunofluorescence assay.	Minari et al. (2011)
	*Botryosphaeria rhodina*	ExP (Botryosphaeran)	Herpes simplex virus type I (HSV-1)-infected Vero cell lines	Botryosphaeran exhibited antiviral activity with IC_50_ values ranging from 2.4 to 3.0 μg/mL against acyclovir-sensitive HSV-1 and from 2.7 to 7.3 μg/mL against acyclovir-resistant HSV-1.	Sacchelli et al. (2019)
	*Lasiodiplodia theobromae*	ExP (Lasiodiplodan)	HSV-1-infected BALB/c mice	Lasiodiplodan inhibited more than 80% of HSV-1 infection across various treatment approaches, including virucidal activity, adsorption inhibition, and post-adsorption effects, while demonstrating preventive effects and inhibiting both DNA and protein synthesis, even at low concentrations.	Wouk et al. (2022)
	*Lentinula edodes*	ExP (Lentinan)	Infectious hematopoietic necrosis virus (IHNV)-infected epithelioma papulosum cyprinid cell lines	At a concentration of 100 μg/mL, lentinan exhibited antiviral activity against IHNV, with inhibition rates ranging from 39.60% to 82.38%, while simultaneously suppressing the expression of three pro-inflammatory cytokines (TNF-*α*, IL-2, and IL-11) and enhancing the expression of two interferons (IFN-1 and IFN-*γ*).	Ren et al. (2018)
		ExP (Lentinan)	Hepatitis B virus (HBV)-infected HepAD38 and HepG2 cell lines	Lentinan at a concentration of 50 μg/mL enhanced the inhibitory effect of Lamivudine (10 μmol/L) on HBV DNA replication.	Jiao et al. (2018)
	*Pleurotus ostreatus*	ExP (Pleuran)	Ninety patients over the age of six diagnosed with herpes simplex	Systemic administration of pleuran effectively reduced the duration of herpes simplex symptoms and decreased the duration and severity of respiratory symptoms compared to the placebo group over a 120-day period, with no adverse effects observed during the clinical trial.	Urbancikova et al. (2020)
	*Pleurotus pulmonarius*	EnP	Influenza A virus-infected Madin-Darby canine kidney (MDCK) cell lines	EnP exhibited moderate antiviral activity against the influenza A (H1N1) strain.	Vlasenko et al. (2020)
Immunomodulating property	*Botryosphaeria rhodina*	ExP (Botryosphaeran)	RAW264.7 macrophage cell lines and Sprague-Dawley rats	Botryosphaeran enhanced NO production, TNF-*α* secretion, and phagocytic activity in RAW 264.7 macrophage cells, while also stimulating mitogen-induced lymphoblastogenesis in the spleens of experimental rats at doses of 1.25 and 12.5 mg/kg body weight.	Weng et al. (2011)
	*Cordyceps militaris*	EnP	RAW264.7 macrophage cell lines	EnP at a concentration of 200 µg/mL enhanced the secretion of NO, TNF-*α*, and IL-6 by activating MAPKs and NF-κB signaling pathways.	He et al. (2020a)
	*Flammulina velutipes*	EnP	RAW264.7 macrophage cell lines	EnP at a concentration of 1,000 µg/mL stimulated an increase in NO, IL-6, and TNF-*α* secretion.	Ye et al. (2020)
		EnP	Mouse B lymphocytes	EnP at a concentration of 200 μg/mL enhanced IL-10 production and elevated IgG and IgM levels via the ERK1/2 and NF-κB signaling pathways.	Wang et al. (2018)
	*Ganoderma atrum*	EnP	Cyclophosphamide-induced immunosuppressed mice	Oral administration of EnPS at a dose of 25, 50, and 100 mg/kg body weight enhanced the secretion of TNF-*α* and IL-12 levels, promoted T and B cell survival, and ameliorated ROS generation and apoptosis.	Li et al. (2017b)
	*Ganoderma lucidum*	EnP	Cyclophosphamide-induced immunosuppressed mice	Oral administration of EnP at a dose of 250 mg/kg body weight increased IgA levels, activated hematopoiesis, and protected the spleen and thymus.	Li et al. (2020a)
	*Grifola frondosa*	EnP	RAW264.7 macrophage cell lines	EnP at a concentration of 1,000 µg/mL increased the secretion of NO, TNF-*α*, and IL-1*β* to 40.74 µmol/mL, 81.84 pg/mL, and 229.07 pg/mL, respectively, through the TRL4 signaling pathway.	Mao et al. (2015)
		EnP	Heps tumor-bearing mice	Oral administration of EnP at a dose of 54 mg/kg body weight increased the weight of the thymus and spleen and enhanced the secretion of TNF-*α*, IL-2, and NO.	Mao et al. (2016)
	*Hericium erinaceus*	EnP	Human monocytic THP-1 cell lines	EnP at a concentration of 50 μg/mL enhanced the secretion of TNF-*α*, IL-1*β*, and IL-6 and stimulated lymphocyte proliferation.	Wu et al. (2019)
	*Inonotus obliquus*	EnP	Human T lymphadenoma jurkat tumor-bearing mice	Oral administration of EnP at a dose of 80 mg/kg body weight increased the secretion of TNF-*α*, IL-2, IL-6, and IL-12 to 174.29 pg/mL, 63.69 pg/mL, 79.58 pg/mL, and 52.79 pg/mL, respectively, and enhanced macrophage phagocytosis.	Chen et al. (2015)
	*Lentinula edodes*	ExP (Lentinan)	RAW264.7 macrophage cell lines and cyclophosphamide induced immunosuppressed mice	At a concentration of 200 μg/mL, lentinan enhanced macrophage phagocytic capacity, improved spleen and thymus indices, stimulated lymphocyte proliferation and regulated the proportions of CD4+ and CD8+ T cells in experimental mice.	Wang et al. (2020b)
	*Pleurotus eryngii*	EnP	RAW264.7 macrophage cell lines	EnP at a concentration of 100 μg/mL increased the secretion of NO, TNF-*α*, IL-1, and IL-6 by 379.9%, 25.4%, 42.9%, and 20.4%, respectively, through the activation of MAPKs and NF-κB signaling pathways.	Xu et al. (2016)
Prebiotic property	*Cantharellus cibarius*	EnP	Rats model	EnP supplementation resulted in the highest *Lactobacillus* counts (1.84 × 10^9^ CFU/mL) and the lowest *Clostridium* counts (0.03 × 10^9^ CFU/mL) in the colonic contents of rats, highlighting its potential to modulate gut microbiota composition effectively.	Uthan et al. (2021)
	*Cordyceps militaris*	ExP	Prebiotic activity assay	ExP demonstrated growth-promoting effects on probiotic strains, including *Lactobacillus rhamnosus, Lac. paracasei, Lac. casei, Lac. acidophilus, Lac. plantarum*, and *Bifidobacterium longum* and *Bi. adolescentis*, exhibiting a performance comparable to that of inulin.	Kang et al. (2022)
	*Ganoderma lucidum*	EnP	High-fat diet-induced obese mice	EnP demonstrated modulation of the gut microbiota by increasing the populations of *Bifidobacterium choerinum* and *Bacteroides chinchillae*, improving gut barrier function, and enhancing the production of short-chain fatty acids (SCFAs) in the gastrointestinal system of experimental mice.	Sang et al. (2021)
		EnP	Azoxymethane and dextran sodium sulfate -induced colitis mice	EnP ameliorated microbiota dysbiosis by increasing the abundance of *Bifidobacterium* and *Lactobacillus*, reducing the populations of *Lachnoclostridium*, *Oscillibacter*, *Desulfovibrio*, *Alistipes*, and *Parasutterella*, while also enhancing the production of short-chain fatty acids and alleviating endotoxemia.	Guo et al. (2021)
	*Lentinus polychrous*	EnP	Prebiotic activity assay	EnP demonstrates strong potential to enhance the growth of probiotics, such as *Limosilactobacillus fermentum* and *Lacticaseibacillus rhamnosus*, while also serving as effective cryoprotectants in freeze-drying, as shown by the over 70% cell survival of *L. fermentum* after 90 days of storage at 4°C.	Panya et al. (2024)
	*Lentinus squarrosulus*	EnP	Prebiotic activity assay	EnP demonstrates strong potential to enhance the growth of probiotics, such as *Limosilactobacillus fermentum* and *Lacticaseibacillus rhamnosus*, while also serving as effective cryoprotectants in freeze-drying, as shown by the over 70% cell survival of *Lim. fermentum* after 90 days of storage at 4°C.	Panya et al. (2024)
	*Lepista sordida*	EnP	Prebiotic activity assay	EnP, utilized as a carbon source, enhanced acid production, significantly increased the biomass of *Lactobacillus casei*, and demonstrated probiotic activity surpassing that of inulin, highlighting its superior efficacy as a prebiotic.	Wang et al. (2022a)
	*Ophiocordyceps dipterigena*	ExP	Chickens	The combination of ExP acting as a prebiotic, and *Lactobacillus acidophilus* as a probiotic, demonstrated promising potential as a novel dietary supplement for chickens, with the presence of *Lac. acidophilus* successfully detected in the fecal samples of chickens fed a diet containing both components, indicating effective colonization and synergistic benefits.	Prathumpai et al. (2015)
	*Pleurotus ostreatus*	EnP	High-fat diet-induced obese mice	EnP had a beneficial impact on the gut microbiota by increasing the abundance of *Oscillospira*, *Lactobacillus*, and *Bifidobacterium*, while reducing the populations of *Bacteroides* and *Roseburia*.	Hu et al. (2022a)
	*Trametes versicolor*	ExP	Prebiotic activity assay	ExP effectively stimulated the growth of probiotic strains, including *Lactiplantibacillus plantarum, Lactobacillus gasseri, Lactobacillus acidophilus, Lacticaseibacillus casei*, and *Limosillactobacillus reuteri*, highlighting its potential as a prebiotic ingredient.	Angelova et al. (2022)

### 9.1 Antidiabetic property

Diabetes, a non-communicable disease characterized by chronic hyperglycemia, is classified into Type 1 and Type 2. Type 1 occurs when the pancreas fails to produce insulin, while Type 2, the most common form, results from the body’s inability to effectively use insulin, often linked to factors like being overweight and lack of physical activity (American Diabetes Association, 2009). Natural supplements derived from plants and fungi, along with medications used to treat diabetes, can help control blood glucose levels (Liu et al., 2022a). According to previous research, fungal polysaccharides could beneficially affect people with diabetes by inhibiting glucose absorption efficacy, gastrointestinal viscosity, inhibition of *α*-amylase and *α*-glucosidase activity to control hyperglycemia, improving pancreatic *β*-cell mass, and enhancing insulin signaling (Jovanović et al., 2021; Fu et al., 2022; Liu et al., 2022a; Figueroa et al., 2023; Ji et al., 2023; Li et al., 2023; Yu et al., 2024). Generally, Streptozotocin-induced diabetic mice are frequently used to assess a compound’s ability to reduce blood glucose levels. This method yields consistent results, demonstrating the compound’s capability to lower blood glucose. Several previous studies demonstrated polysaccharides from *Aur*. *auricula*, *Aur*. *polytricha*, *Co*. *taii*, *D*. *indusiate*, *G*. *frondose*, *Pl*. *eryngii*, *Pl*. *geesterani*, *Pl*. *ostreatus*, and *Su. luridus* can effectively reduce blood glucose levels in Streptozotocin-induced diabetic mice (Duobin et al., 2013; Zhang et al., 2018a; Chen et al., 2019; Liu et al., 2019; Xiang et al., 2021) (**Table 6**). These findings indicate that fungal polysaccharides could be used as supplements for diabetes. However, future studies must evaluate the effectiveness of these fungal polysaccharides in diabetic patients to assess their true efficacy, safety, and potential side effects. If these polysaccharides can be developed into a treatment for diabetes, they could greatly benefit both medical practice and pharmacology by offering patients a more effective and safer treatment option. Moreover, the development of drugs based on fungal polysaccharides could offer new opportunities for treating diabetes.

### 9.2 Antilipidemic property

Hyperlipidemia is a condition characterized by elevated levels of total cholesterol (TC), triglycerides (TG), and low-density lipoprotein cholesterol (LDL-C) in the blood, exceeding established thresholds. It is a significant risk factor for various disorders, including diabetes, hypertension, stroke, and cardiovascular disease. In addition to conventional medications, dietary supplements provide an alternative for individuals with hyperlipidemia, with fungal polysaccharides emerging as a promising option due to their antilipidemic properties (De Costa and Park, 2017). Prior research employing mouse models has frequently shown that fungal polysaccharides can successfully reduce blood levels of LDL-C, TC, and TG (**Table 6**) (Miranda-Nantes et al., 2011; Zeng et al., 2013; Wang et al., 2019b; Ge et al., 2022). Although animal studies have demonstrated the efficacy of fungal polysaccharides in significantly reducing TC, TG, and LDL-C, further clinical trials in patients with hyperlipidemia are required to confirm their effectiveness.

### 9.3 Antibacterial property

The antibacterial effects refer to the ability of substances or materials to inhibit the growth of various bacteria through mechanisms such as disrupting the bacterial cell wall, inhibiting enzymes essential for bacterial growth, or preventing bacteria from adhering to host cells (Di Martino, 2022). Previous studies have focused on exploring the antibacterial properties of fungal polysaccharides, revealing that these polysaccharides have the potential to inhibit the growth of human pathogenic bacteria, including genera *Bacillus*, *Escherichia*, *Klebsiella*, *Listeria*, *Micrococcus*, *Pseudomonas*, *Proteus*, *Shigella*, *Salmonella*, and *Staphylococcus*, as well as methicillin-susceptible *Staphylococcus aureus* (Skalicka-Woźniak et al., 2012; Mahendran et al., 2013; Osińska-Jaroszuk et al., 2014; Wan-Mohtar et al., 2016; Ayimbila et al., 2021) (**Table 6**). The effectiveness of these polysaccharides varies depending on the type of fungal polysaccharide used. Thus, fungal polysaccharides exhibit promising bacterial properties and show potential for development as components in products for preventing and treating bacterial infections. However, further research is required to confirm their efficacy and safety for human use.

### 9.4 Antioxidant property

Free radicals, generated in the body through normal metabolism or external factors like UV radiation, pollution, or chemicals, are unstable and stabilize by stealing electrons from other molecules. They can lead to oxidative stress, which damages cells and tissues, contributing to various diseases such as heart disease, cancer, and accelerated aging (Phaniendra et al., 2015). However, the human body has compounds that protect against oxidative stress, called antioxidants, which help neutralize free radicals and reduce the damage they may cause. Antioxidants can be obtained from external sources such as food and supplements (Pham-Huy et al., 2008). Interestingly, certain fungi can produce polysaccharides with antioxidant properties, adding another potential source of these beneficial compounds (Bai et al., 2022). Previous studies have reported that fungal polysaccharides possess antioxidant properties, as demonstrated using various methods, including ABTS, DPPH, hydroxyl radical, and superoxide radical assays (**Table 6**) (Liu et al., 2018; Si et al., 2018; Ayimbila et al., 2021; Deng et al., 2021; Lu et al., 2022; Tabibzadeh et al., 2022). These assays help evaluate the potential of fungal polysaccharides in protecting cells from damage caused by free radicals, providing researchers with deeper insights into their role in mitigating cellular damage. However, one concern with fungal polysaccharides is the variability in their antioxidant efficacy. Thus, optimizing cultivation conditions, standardizing extraction methods, using specific fungal strains, and implementing rigorous testing and quality control can help control and reduce the variability in the antioxidant efficacy of fungal polysaccharides.

### 9.5 Anticancer property

Cancer is currently one of the leading causes of death worldwide. Despite significant advancements in research and treatment, including immunotherapy, targeted therapy, and proton therapy, challenges persist. Certain types of cancer remain complex and show limited response to treatment. In addition, side effects from therapies such as chemotherapy and radiation can negatively impact a patient’s quality of life (Christyani et al., 2024; Tufail et al., 2024). The alternative therapeutic strategies are being explored, including the potential of polysaccharides derived from fungi. Research has increasingly focused on the anticancer properties of fungal polysaccharides, which have shown promise in combating cancer through various mechanisms. Current studies have shown that fungal polysaccharides possess anticancer properties, with experiments conducted both *in vitro* using cell lines and *in vivo* in animal models (**Table 6**). However, while they induce apoptosis in cancer cells and inhibit cancer cell growth, the results obtained are still in the preliminary stages and have limitations (Mao et al., 2016; Su et al., 2018; Geraldelli et al., 2020; Udchumpisai and Bangyeekhun, 2020; Wu et al., 2022b; Abdala-Díaz et al., 2024). Although initial findings show promising potential, further long-term studies and clinical trials in humans are necessary to confirm the effectiveness and safety of using fungal polysaccharides as a therapeutic approach to cancer treatment.

### 9.6 Antiviral property

Developing antiviral substances holds significant importance for advancements in medicine and public health (Gonçalves et al., 2021). Antiviral properties are one of the key characteristics of fungal polysaccharides that have been extensively studied. Research has demonstrated that fungal polysaccharides can inhibit viral replication, including against herpes simplex virus type I (Sacchelli et al., 2019), hepatitis B virus (Jiao et al., 2018), and influenza A virus (Vlasenko et al., 2020) (**Table 6**). Although current research has yet to definitively demonstrate that fungal polysaccharides inhibit several viruses, ongoing studies are required to fully realize their potential in antiviral therapeutics. Further studies should address important constraints, including their narrow spectrum, unknown mechanisms, low bioavailability, and the need for additional clinical evidence.

### 9.7 Immunomodulating property

Fungal polysaccharides are known for their immunomodulatory properties, with low toxicity, high molecular mass, and diverse branching structures that may trigger appropriate immune responses in humans (Zhao et al., 2020; Yin et al., 2021). Fungal polysaccharides possess notable properties in modulating and enhancing immune system function. The key mechanism involves activating immune cells, such as macrophages, and stimulating cytokine secretion, which further supports immune system activity (**Table 6**). These polysaccharides hold the potential for development into immune-boosting supplements or therapeutic agents to prevent and alleviate the effects of infections, chronic diseases, or immune deficiencies (Murphy et al., 2023a, b). However, most of the research to date has been limited to *in vitro* and *in vivo* studies, which may not be sufficient for the development of clinical-grade therapeutics. Therefore, further clinical studies in humans are necessary to confirm the effectiveness of fungal polysaccharides in immune modulation, as well as to evaluate any potential side effects, ensuring safety and reliability for practical use.

### 9.8 Prebiotic property

Prebiotic property is another important characteristic of polysaccharides, as previous research has shown that polysaccharides from edible fungi are resistant to human digestive enzymes and serve as a crucial energy source for the gut microbiome, promoting the growth of beneficial bacteria (Uthan et al., 2021; Zhao et al., 2023b). *Bifidobacterium*, *Lacticaseibacillus*, and *Lactobacillus* are key probiotic bacteria in the human gastrointestinal system, playing an essential role in promoting intestinal health, supporting immune function, and maintaining the balance of gut microbiota (Wang et al., 2022b; Panya et al., 2024). Recent studies have highlighted the potent prebiotic properties of fungal polysaccharides, which can enhance the growth of probiotics both *in vitro* and in animal models (**Table 6**). These findings suggest that fungal polysaccharides hold significant potential for development as functional ingredients in prebiotic products that support gastrointestinal health (Barcan et al., 2024). Despite their potential as prebiotic agents, fungal polysaccharides have a number of limitations that require more further research and development. Unlocking their full potential as functional components in prebiotic products will require increasing their bioavailability, understanding their mechanisms of action, establishing clinical evidence, and resolving issues with cost and production scalability.

## 10 Applications for fungal polysaccharides

**Figure 5:**
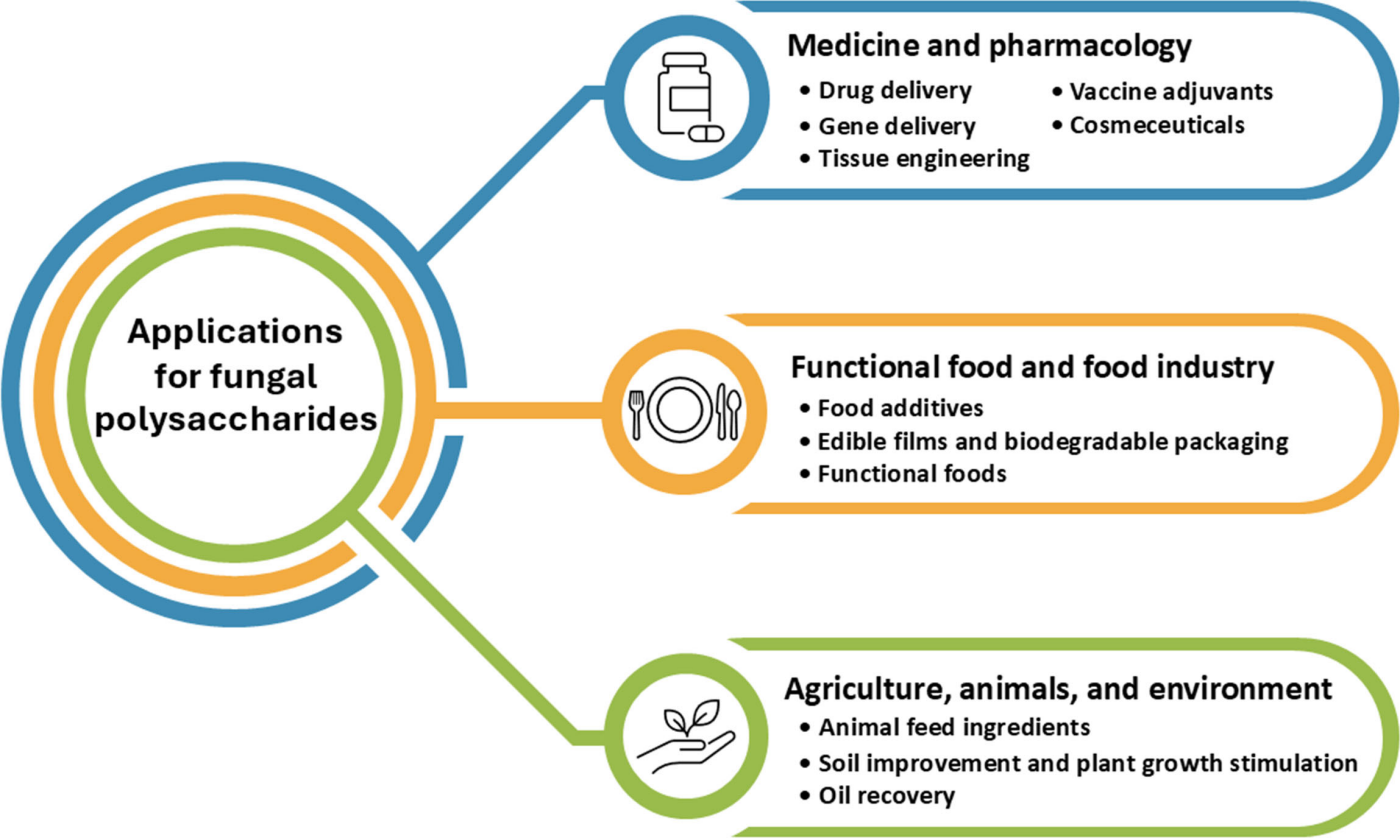
Applications of fungal polysaccharides in medicine, pharmacology, food industry, agriculture, animals, and environment.

This research presents the application of polysaccharides derived from fungi in the fields of medicine, pharmacology, food, agriculture, animal, and environmental applications as shown in **Figure 5**.

### 10.1 Applications in medicine and pharmacology

#### 10.1.1 Drug delivery

Considerable advancements have been made in drug delivery systems, enhancing the precision of drug release, stability against degradation, and extending the duration of drug action. Innovations such as microcapsules, nanoparticles, and polysaccharide-based coatings have been developed to improve the accuracy of drug targeting (Ezike et al., 2023). The incorporation of polysaccharide-based coatings in drug delivery systems offers several advantages, including safety, stability, controlled release, biodegradability, and natural origin, making these materials highly compatible. This versatility, especially when combined with other delivery platforms, broadens the potential applications of polysaccharides in drug administration (Visan and Cristescu, 2023). Fungal polysaccharides, particularly pullulan, play a crucial role in drug delivery due to their unique beneficial properties. As noted by Thakur et al. (2023), pullulan-based polymeric drug delivery systems offer a pioneering approach for respiratory disease treatment, featuring a sustained release profile, biocompatibility, non-immunogenicity, chemical modifiability, and superior permeability through lung mucosa compared to other polysaccharides. Gehrcke et al. (2022) have developed a bilayer film composed of pullulan, encapsulating silibin-loaded nanocapsules for the treatment of atopic dermatitis. *In vitro*, results demonstrated that pullulan enhances the film’s adhesion to skin tissue, and the film possesses antioxidant properties without inducing hemolysis. Interestingly, *in vivo* experiments, the film regulated inflammation and oxidative parameters as effectively as, or even better than, silibinin solution and hydrocortisone, which are conventional treatments for atopic dermatitis. In addition, Balasso et al. (2017) used pullulan in a bioconjugate specifically designed for targeted drug delivery to hepatocellular carcinoma. The results indicated that the PreS1-Pullulan-Doxorubicin polymer enhanced anticancer efficacy against HepG2/SERPINB3 cells twofold compared to the control pullulan-Doxorubicin. This finding highlights the potential of enhancing polysaccharides as selective therapeutic agents for hepatocellular carcinoma by promoting targeted accumulation within cancerous liver cells. Furthermore, research has been conducted on drug delivery using schizophyllan, which forms stearic acid-schizophyllan micelles, a novel type of drug carrier (Negahban et al., 2021). Paclitaxel loaded into these micelles exhibited higher anticancer activity against MCF-7 cells compared to free paclitaxel. Notably, schizophyllan also possesses unique anti-inflammatory and immune-enhancing properties, which could potentially synergize with the therapeutic effects of the loaded drug.

#### 10.1.2 Gene delivery

Gene delivery refers to the process of delivering genetic materials, such as DNA or RNA, to various cells or tissues in the body to modify, enhance, or alter gene function. This technique is widely used in genetic research, vaccine development, gene therapy, and studies related to various diseases (Sung and Kim, 2019). Gene delivery can be achieved through several methods, including the use of viruses that have been modified to be non-pathogenic, such as adenoviruses, lentiviruses, and adeno-associated viruses (AAV), to deliver genetic materials into cells. Additionally, non-viral approaches utilize nanocarriers made from synthetic materials or natural substances to facilitate gene delivery (Pan et al., 2021). These include polymeric nanoparticles or polysaccharides, providing alternative methods for introducing genetic material without the use of viruses (Li et al., 2018b). Previous research by Kang et al. (2010) incorporated the polysaccharide pullulan into polyethyleneimine (PEI) and small interfering RNA (siRNA) to create a pullulan-modified PEI/siRNA complex for targeted delivery to the liver, comparing it with the PEI/siRNA complex. After the injection into mice, siRNA was detected using fluorescence techniques. The results revealed that the PEI-pullulan/siRNA complex exhibited higher fluorescence in the liver compared to the PEI/siRNA complex, while the PEI/siRNA complex demonstrated greater fluorescence in the lungs. The PEI-pullulan/siRNA complex significantly reduced mouse mortality and could serve as an effective, low-toxicity strategy for delivering siRNA to the liver. Similar to other targeted delivery systems for plasmid DNA (pDNA) and siRNA directed at folate receptors on cancer cells, Wang et al. (2014a) synthesized an enhanced gene delivery carrier using folate-polyethyleneimine-modified pullulan for the delivery of pDNA and siRNA. The results indicated that the folate-polyethyleneimine-modified pullulan effectively encapsulates pDNA and siRNA, with efficient delivery occurring through the folate receptor. This suggests that folate-polyethyleneimine-modified pullulan is a promising, safe, and precise gene delivery system suitable for targeting cancer cells. Additionally, the study by Liu et al. (2014) developed an innovative nanoparticle system with a core-shell structure, utilizing pullulan and poly(*β*-amino) ester (PBAE) conjugated to methotrexate (anti-cancer drug) and green fluorescent protein (pEGFP). This methotrexate-pullulan/PBAE/pEGFP nanoparticle was specifically designed for the targeted delivery of gene and chemotherapeutic agents to liver cancer cells. This nanoparticle exhibited strong hepatoma-targeting capability, primarily accumulating in liver cancer cells within 24 hours post-intravenous injection. It successfully facilitated the co-delivery of gene and chemotherapy agents to tumor sites at both the cellular and animal levels, offering a promising approach to liver cancer treatment.

#### 10.1.3 Tissue engineering

Tissue engineering is an emerging field that plays a crucial role in promoting the regeneration of damaged tissues that are unable to self-repair. It enhances the body’s natural healing potential, supporting recovery in a way that complements the patient’s healing process. Injured tissue repair can be stimulated through the use of synthetic polymer scaffolds, which serve as structural supports that facilitate efficient tissue regeneration (Singh et al., 2016). In a prior study by Schlaubitz et al. (2014), pullulan combined with dextran and reinforced with nanocrystalline hydroxyapatite was developed into highly porous scaffold beads to evaluate cellular growth and new bone formation in a rat model. The findings indicated effective cellular penetration into the scaffold spaces and new tissue formation around the beads. Additionally, calcium and mineral deposition within the structure increased over time, with no inflammation observed at the implantation site. Thangavel et al. (2020) used pullulan gel to evaluate its wound-healing efficacy on open excision wounds made on the dorsum of rats. The study found that pullulan gel significantly stimulated collagen, hexosamine, protein, and DNA synthesis compared to the untreated and povidone-iodine ointment-treated groups, demonstrating its potential as a wound-healing agent. The effects of *β*-glucan from *S. cerevisiae* on wound healing in venous ulcers in humans were evaluated by Medeiros et al. (2012) through histopathological analysis. The findings revealed that β-glucan significantly accelerated wound healing by promoting epithelial hyperplasia, stimulating angiogenesis, increasing plasmocyte numbers, and enhancing fibroblast proliferation.

#### 10.1.4 Vaccine adjuvants

Vaccine adjuvants are substances or components added to vaccines to enhance the immune response’s effectiveness. Typically, vaccines contain immunogenic agents such as antigens; however, the incorporation of adjuvants intensifies and prolongs the immune stimulation. Therefore, the use of adjuvants in vaccines enables the development of more effective vaccines and enhances their ability to prevent various diseases more effectively (Zhao et al., 2023c). A study by Zhang et al. (2017) found that lentinan, tremella, pachymaran, and their combination with the H1N1 influenza vaccine in mice enhanced the vaccine’s efficacy. These findings suggest that fungal polysaccharides could help improve the effectiveness of influenza vaccines. He et al. (2020b) used calcium carbonate-lentinan loaded with the H5N1 antigen to develop an adjuvant for the H5N1 vaccine, aimed at preventing the avian influenza virus. After injection into mice, the calcium carbonate-lentinan/H5N1 complex significantly enhanced the expression of MHC-II and CD86 in dendritic cells from lymph nodes. It also unexpectedly led to elevated hemagglutination inhibition (HI) titers and stimulated the secretion of IgG subtypes (IgG1 and IgG2b), along with T-helper-associated cytokines (TNF-*α*, IFN-*γ*, and IL-4) in vaccinated mice. Additionally, Liu et al. (2019) developed a vaccine by encapsulating polysaccharides from *G. lucidum* and inactivated porcine circovirus type II (PCV-II) into liposomes. When administered to mice, this vaccine induced stronger PCV-II-specific immune responses, including higher titers of PCV-II-specific IgG antibodies, increased cytokine levels, and splenocyte activation, compared to other single-component formulations.

#### 10.1.5 Cosmeceuticals

Cosmeceuticals are products that combine the properties of cosmetics and pharmaceuticals to enhance the effectiveness of skincare and beauty treatments. These products typically contain active ingredients that nourish or treat the skin, such as vitamins, minerals, antioxidants, and natural substances. They are designed to improve skin health, prevent deterioration, and effectively reduce the appearance of wrinkles (Singh et al., 2024). Kanlayavattanakul and Lourith (2023) found that the addition of polysaccharide extract from *Co. militaris* to a skincare cream formulation increased the cream’s stability. A study by Woźniak et al. (2023) revealed that polysaccharide extract from *Tre. fuciformis* serves as a natural alternative to hyaluronic acid in cosmetic formulations, significantly improving skin hydration without causing irritation or erythema, as confirmed by dermatological evaluations. A study by Sangthong et al. (2022) demonstrated that a cosmetic gel cream containing polysaccharides from *V. volvacea* could enhance skin hydration, elasticity, and firmness, while reducing skin roughness, dryness, wrinkles, and melanin content, with no cytotoxic effects on human dermal fibroblasts. Additionally, a study conducted by Jesenak et al. (2016) demonstrated that the application of Imunoglukan P4H^®^ cream, containing *β*-glucan (pleuran isolated from *Pl. ostreatus*), significantly reduced the frequency and severity of atopic dermatitis in patients.

### 10.2 Applications in functional food and food industry

#### 10.2.1 Food additives

Fungal polysaccharides can be applied in the food industry as additives to improve food quality, such as gelling agents, stabilizers, thickeners, or texture modifiers. These polysaccharides do not affect the taste of food, which is a critical aspect to consider in food products, while also enhancing sensory properties. Furthermore, polysaccharides help extend the shelf life of food (Jindal and Singh Khattar, 2018). Pullulan has been used in the food industry for over 20 years and has been recognized as Generally Recognized as Safe (GRAS) in the USA (Oğuzhan and Yangılar, 2013). This is due to its characteristics as a colorless, odorless, edible polysaccharide that is non-toxic and non-carcinogenic (Prajapati et al., 2013). Pullulan has been utilized as a thickening agent in foods such as soups, sauces, and various beverages (Yatmaz and Turhan, 2012). According to Singh et al. (2019), pullulan has been used as a stabilizer and texture modifier in mayonnaise, and it has also been employed to enhance the adhesion of nuts in cookies. Furthermore, pullulan has low viscosity and remains stable when exposed to sodium chloride and heat, making it an ideal thickening agent for food products with high salt concentrations, such as barbecue sauces, soy sauces, pickled fruits, and vegetables. In addition, scleroglucan can serve as a gelling agent, stabilizer, or thickener, offering an alternative to xanthan gum in the food industry. It improves the quality of heat-treated and frozen foods, such as steamed foods, rice crackers, Japanese cakes, and bread (Jindal and Singh Khattar, 2018). Pleuran and lentinan can be used to produce hydrogels for yogurt products without affecting the flavor or texture while enhancing health benefits (Vetter, 2023).

#### 10.2.2 Edible films and biodegradable packaging

Fungal polysaccharides can be utilized in the production of edible or biodegradable films (Cazón et al., 2017). Pullulan is widely used in the fabrication of edible films due to its strength, flexibility, and excellent oxygen barrier properties, which prevent food oxidation from air exposure (Oğuzhan and Yangılar, 2013). Edible films made from pullulan have also been employed in film formation and packaging for various foods, including vegetables, fruits, grains, snacks, and dried goods (Singh et al., 2016, 2019). Previous studies have shown that pullulan, when combined with other substances such as chitosan, alginate, or shellac, can create films with oxygen, water, and UV protection, making them suitable for food packaging (Zhou et al., 2021; Mugnaini et al., 2024; Tang et al., 2025). Additionally, pullulan has been incorporated with propolis to develop antifungal food packaging, which protects against pathogenic fungi, thus preventing fungal growth in food and extends food shelf life (Gniewosz et al., 2022). A study by Viñarta et al. (2013) revealed that scleroglucan derived from *Scl. rolfsii* demonstrated superior performance, particularly in enhancing pseudoplastic behavior, and showed excellent compatibility with corn starch, xanthan, pectin, and carboxymethylcellulose, making it highly suitable for applications in the food industry. Beyond pullulan, scleroglucan, and schizophyllan can be used to form edible films for dietary supplement packaging due to their chemical stability and natural biodegradability (Rahman et al., 2021; Ghosh et al., 2022).

#### 10.2.3 Functional foods

Edible mushrooms are recognized as health-promoting foods for humans. In recent years, bioactive polysaccharides extracted from edible mushrooms have been developed into functional foods that are more easily and rapidly absorbed by the body, resulting in faster bioactivity. In addition to functional foods for human health, polysaccharides from mushrooms can also be used as animal feed (Osińska-Jaroszuk et al., 2020). *β*-glucan, a polysaccharide derived from mushrooms, is well-known for its diverse biological and pharmacological properties, including immunomodulatory, antioxidant, antimicrobial, anticancer, cardioprotective, and hepatoprotective activities. *β*-glucan from mushrooms can enhance both innate and cell-mediated immune responses. It has demonstrated varying degrees of antitumor activity in humans due to differences in structure, water solubility, size, and molecular mass (Khan et al., 2018). Furthermore, previous studies have shown that polysaccharides from several mushrooms, e.g. *G. lucidum*, *Le. edodes*, *Tre. fuciformis*, *Au. pullulans*, *Pleurotus* spp., and *Ag. bisporus* (Cheng et al., 2011; Giannenas et al., 2011; Delzenne and Bindels, 2015) exhibit prebiotic properties, promoting the growth of beneficial bifidobacteria, a probiotic in the human gut.

Currently, polysaccharide extracts from mushrooms are commercially available as dietary supplements and functional foods. For instance, Immune-Assist™ Critical Care Formula, produced by Aloha Medicinals Inc., contains extracts from various mushrooms, including *Ag. blazei* (58.5% *β*‐glucan), *Co. sinensis* (30% *β*‐glucan), *Gr. frondosa* (28% *β*‐glucan), *Le. edodes* (40% *β*‐glucan, lentinan, and α‐glucan), *Tr. versicolor* (40% *β*‐glucan), and *G. lucidum* (40% *β*‐glucan and triterpenoids). This product has been shown to reduce the side effects of chemotherapy and radiotherapy in cancer patients. Super Reishi^®^, a *β*‐glucan extract from *G. lucidum* produced by Mushroom Wisdom Inc., is known for modulating and supporting multiple bodily systems, including the heart, lungs, liver, nervous system, and brain. Maitake^®^, developed by Pharmaceutical Mushrooms Inc., is a *β*‐glucan-based product derived from *Gr. frondosa* that demonstrates strong immunomodulatory effects, notably enhancing T-cell production, and is specifically recommended for managing immunodeficiency conditions. Fine‐Agaricus^®^ Gold, produced by FineCo. Ltd. is a 100% polysaccharide extract from *Agaricus* that has demonstrated efficacy against various cancers through immune system enhancement, while also balancing physiological functions and providing benefits for the treatment of chronic diseases. Additionally, Transfer Factor Plus^®^ Tri‐Factor^®^ Formula, produced by Product 4life Inc., contains extracts from *Le. edodes*, *Gr. frondosa*, and *Cordyceps* sp., comprising *β*‐glucans, hexaphosphate inositol, *β*‐sitosterol, and an extract of olive leaves. This formula has been shown to stimulate the immune system by enhancing the activity of NK cells in the body (Morris et al., 2016).

### 10.3 Applications in agriculture, animals, and environment

#### 10.3.1 Animal feed ingredients

Polysaccharides from fungi, in addition to being used as functional food for humans, can also be applied as animal feed ingredients to enhance the nutritional value of livestock (Osińska-Jaroszuk et al., 2020). Research by Muthusamy et al. (2013) demonstrated that *β*-glucan derived from *Pl. floridanus* could be incorporated into poultry feed, significantly enhancing the immune response in broiler chickens. Similar to the study by Gao et al. (2024), which found that supplementation of polysaccharides from *G. lucidum* increased HDL-C levels in the serum, reduced TG levels, enhanced antioxidant activity, increased antioxidant enzyme levels in broiler chickens, and contributed to the improvement of gut microbiota composition. In addition, *β*-glucan from *Pl. floridanus* and *Pl. ostreatus* has been used in fish feed to enhance immune function in fish (Dobšíková et al., 2013). Furthermore, *β*-glucan has been combined with milk to serve as a dietary supplement in newly weaned piglets, promoting growth performance and gut health (Mukhopadhya et al., 2019).

#### 10.3.2 Soil improvement and plant growth stimulation

Fungal polysaccharides can be applied in agriculture, particularly ExP, which are mucilaginous and carry ionic charges, allowing them to bind soil particles together and enhance soil fertility (Costa et al., 2018). Previous research introduced the use of ExPs in soil, revealing that ExPs improve the aggregate stability of soil particle aggregation in water, thereby enhancing water retention and increasing soil porosity (Costa et al., 2018; Angulo et al., 2024). ExPs produced by basidiomycetes and *Trichocomacea* can bind soil particles, promoting soil aggregate formation and stability (Daynes et al., 2012; Angulo et al., 2024). Polysaccharides from *G. lucidum* have been shown to increase seed germination rates and seedling heights when combined with chemical fungicides in seed-coating formulations, effectively controlling soil-borne diseases. Furthermore, polysaccharides from *G. lucidum* enhance the expression of genes associated with disease resistance in maize and wheat, enabling their use alongside chemical fungicides in the prevention of maize root rot, wheat root rot, and sharp eyespot diseases (Yang et al., 2022a, b). Application of polysaccharides isolated from the fruiting body of *Pl. ferulae* exhibited antifungal activity against *Rhizoctonia solani*, promoted cucumber plant growth by enhancing root length and fresh weight of cucumber seedlings, and stimulated the activities of enzymes such as superoxide dismutase, peroxidase, and polyphenol oxidase for disease resistance (Yang et al., 2024c).

#### 10.3.3 Oil recovery

Oil recovery refers to the process of increasing the extraction rate of crude oil from underground reservoirs (Jafarinejad, 2017). After conventional extraction methods, substantial amounts of oil often remain trapped within the rock formations, necessitating enhanced techniques such as enhanced oil recovery (EOR). EOR methods utilize chemical agents, thermal injections, or biotechnological solutions to improve the recovery of this residual oil (Rizvi, 2024). Fungal polysaccharides are particularly promising for EOR applications. These polysaccharides can enhance the viscosity of injected solutions, effectively displacing oil trapped within the pore spaces of rock formations, thus facilitating oil movement toward the extraction wellbore. Additionally, fungal polysaccharides exhibit high stability under extreme temperature and pressure conditions, making them well-suited for EOR in the challenging underground environments of oil reservoirs (Xia et al., 2020). For example, scleroglucan can be modified to acquire hydrophobic properties by grafting stearate groups, along with the addition of ionic-sulfonic groups to enhance the viscosity of the compound. This modification alters the adsorption behavior of scleroglucan on oil reservoir rock surfaces, with greater stearate grafting density resulting in increased adsorption (Bakhshi et al., 2017). Schizophyllan is used in EOR processes and has been shown to increase the recovery of crude oil by up to 28% over the residual oil saturation (Joshi et al., 2016). Additionally, the use of pullulan in microbial-enhanced oil recovery experiments with Berea sandstone core samples resulted in a 9.4% increase in the recovery of medium-heavy oil (Elshafie et al., 2017).

## 11 Patent search

**Figure 6:**
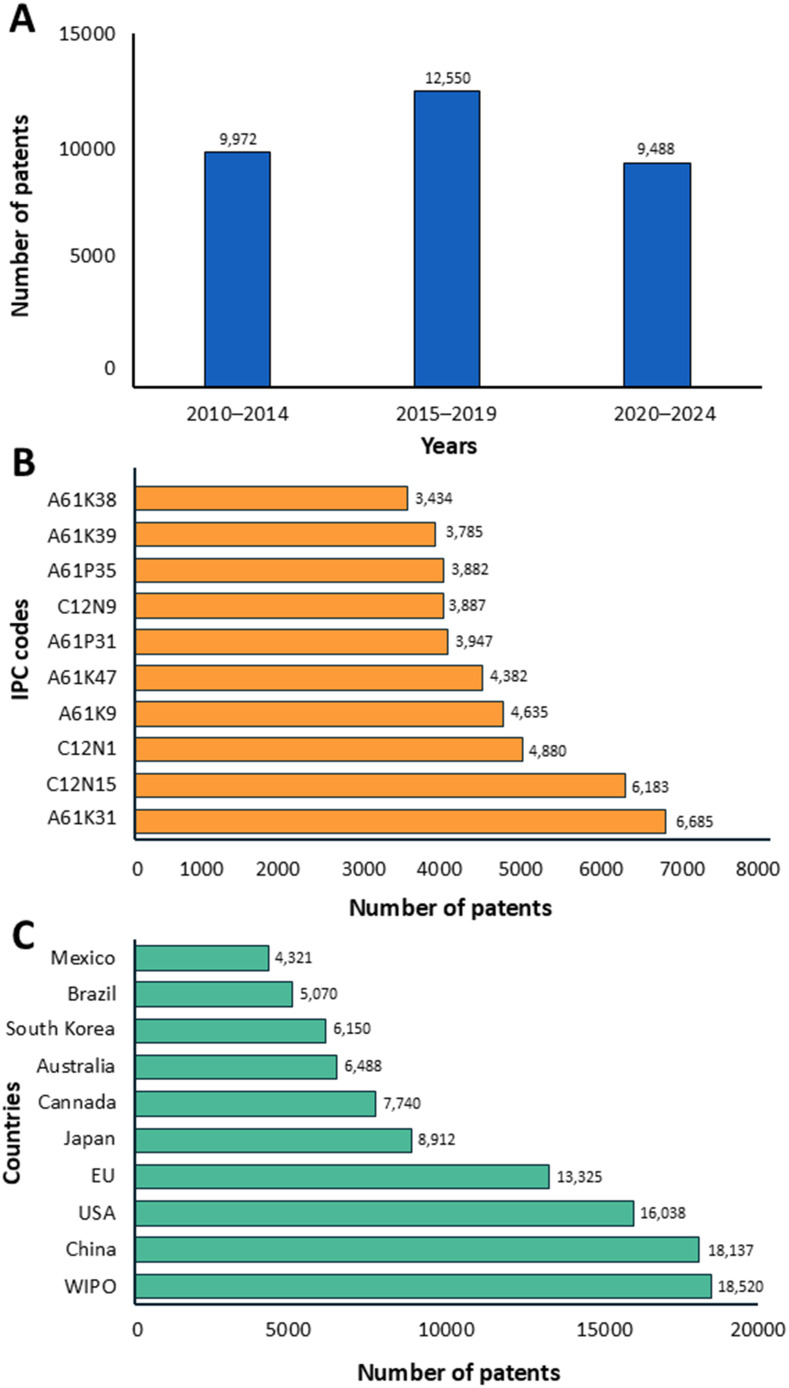
The number of patents **(A)**, the top ten IPC category codes **(B)**, and publication locations **(C)** between 2010 and 2024 of fungal polysaccharides. The search was conducted using the European database Espacenet (accessed on 25 March 2025).

A search for patents using the keyword “fungal polysaccharides” in the Espacenet database (https://worldwide.espacenet.com, accessed 25 March 2025) reveals significant changes in patent filing trends between 2010 and 2024. The data can be categorized into three periods, as presented in **Figure 6A**. The number of patent filings increased significantly from approximately 9,972 between 2010 and 2014 to around 12,550 from 2015 to 2019, indicating substantial growth in interest. However, this trend reversed from 2020 to 2024, with filings decreasing to 9,488. This decline may be influenced by factors such as economic conditions, policy shifts, and evolving technological advancements during that period, as well as delays in research and patent applications resulting from the COVID-19 pandemic.

Based on the International Patent Classification (IPC) codes, the top ten patent classifications for fungal polysaccharides revealed that category A61K31, which covers organic compounds for medicinal and cosmetic preparations, had the highest total with 6,685 patents (**Figure 6B**). This was followed by category C12N15 (genetic modification, vectors, and genetic material manipulation) with 6,183 patents, C12N1 (microorganisms and biotechnological processes) with 4,880 patents, A61K9 (pharmaceutical formulations) with 4,635 patents, and A61K47 (drug carriers and excipients) with 4,382 patents. Other notable categories included A61P31 (anti-infectives), C12N9 (enzymes and proenzymes), A61P35 (antineoplastic agents), and A61K39 (medicinal preparations containing antigens or antibodies). This indicates that categories involving organic compounds for pharmaceutical and cosmetic formulations were particularly popular, reflecting the strong potential of fungal polysaccharides to meet competitive market demands with value-added products. The top ten countries for fungal polysaccharide-related patent filings are shown in **Figure 6C**. The majority of patent filings were made through the World Intellectual Property Organization (WIPO) with 18,520 patents, followed by China with 18,137 patents, the United States with 16,038 patents, and the European Patent Office (EU) with 13,325 patents. Japan ranked fifth with 8,912 patents, followed by Canada (7,740 patents), Australia (6,488 patents), South Korea (6,150 patents), Brazil (5,070 patents), and Mexico (4,321 patents), rounding out the list.

## 12 Conclusions and future perspectives

Fungal polysaccharides are derived from a wide variety of fungal species, including yeasts, filamentous fungi, and mushrooms. The types of polysaccharides vary in composition and structure. To obtain the highest yield of fungal polysaccharides, the processes of fermentation, extraction, precipitation, and purification must be optimized for each fungal species and strain. Each step requires careful consideration.

In recent years, significant advancements have been made in the study of fungal polysaccharides, particularly in their structural characterization, bioactivities, and biotechnological applications. Despite this progress, notable deficiencies remain in our fundamental understanding, especially regarding structure–function and size–function relationships, as well as biosynthetic pathways. Fungal polysaccharides possess diverse biological properties and potential therapeutic benefits. These polysaccharides are used in agriculture, medicine, food, cosmetics, and biotechnology industries. The trend in research on fungal polysaccharides has been growing significantly in recent years. Future research may focus on discovering new fungal species and strains to enhance the optimization of production processes, reduce costs, and support large-scale industrial applications, along with the integration of advanced technologies. Advances in extraction, purification and characterization techniques will further enhance the quality and functional properties of fungal polysaccharides, enabling more precise applications in pharmaceuticals, food, cosmetics, and biotechnology. Moreover, challenges persist in the standardization of extraction and purification methods, which continues to be a major limitation in the field. Although fungal polysaccharides exhibit great potential in medicine and the food industry, their practical applications are still in the experimental and clinical testing stages. Furthermore, continued further research into the biomedical applications of fungal polysaccharides, such as drug delivery systems, immune modulation, and tissue engineering, will expand their impact in healthcare. The biological responses of the human body to fungal polysaccharides are not yet fully understood, highlighting the need for further studies on their safety and health impacts. Finally, to advance both the fundamental understanding and practical applications of fungal polysaccharides, these gaps must be addressed through advanced integrative techniques, including omics (genomics, transcriptomics, proteomics, and metabolomics), CRISPR-based genome editing, bioinformatics, and systems biology, combined with interdisciplinary collaboration.
